# Identification of *Leishmania* Proteins Preferentially Released in Infected Cells Using Change Mediated Antigen Technology (CMAT)

**DOI:** 10.1371/journal.pntd.0000842

**Published:** 2010-10-05

**Authors:** Peter E. Kima, J. Alfredo Bonilla, Eumin Cho, Blaise Ndjamen, Johnathan Canton, Nicole Leal, Martin Handfield

**Affiliations:** 1 Department of Microbiology and Cell Science, University of Florida, Gainesville, Florida, United States of America; 2 Department of Infectious Diseases and Pathology, University of Florida, Gainesville, Florida, United States of America; 3 Department of Oral Biology and Center for Molecular Microbiology, University of Florida, Gainesville, Florida, United States of America; 4 Oragenics, Inc., Alachua, Florida, United States of America; Yale University, United States of America

## Abstract

Although *Leishmania* parasites have been shown to modulate their host cell's responses to multiple stimuli, there is limited evidence that parasite molecules are released into infected cells. In this study, we present an implementation of the change mediated antigen technology (CMAT) to identify parasite molecules that are preferentially expressed in infected cells. Sera from mice immunized with cell lysates prepared from *L. donovani* or *L. pifanoi*-infected macrophages were adsorbed with lysates of axenically grown amastigotes of *L. donovani* or *L. pifanoi*, respectively, as well as uninfected macrophages. The sera were then used to screen inducible parasite expression libraries constructed with genomic DNA. Eleven clones from the *L. pifanoi* and the *L. donovani* screen were selected to evaluate the characteristics of the molecules identified by this approach. The CMAT screen identified genes whose homologs encode molecules with unknown function as well as genes that had previously been shown to be preferentially expressed in the amastigote form of the parasite. In addition a variant of Tryparedoxin peroxidase that is preferentially expressed within infected cells was identified. Antisera that were then raised to recombinant products of the clones were used to validate that the endogenous molecules are preferentially expressed in infected cells. Evaluation of the distribution of the endogenous molecules in infected cells showed that some of these molecules are secreted into parasitophorous vacuoles (PVs) and that they then traffic out of PVs in vesicles with distinct morphologies. This study is a proof of concept study that the CMAT approach can be applied to identify putative *Leishmania* parasite effectors molecules that are preferentially expressed in infected cells. In addition we provide evidence that *Leishmania* molecules traffic out of the PV into the host cell cytosol and nucleus.

## Author Summary

*Leishmania* are intracellular parasites that reside within parasitophorous vacuoles (PV) in phagocytes. From within these compartments parasites control the host cell's responses to multiple stimuli. There is limited knowledge of the molecules that *Leishmania* parasites elaborate in the host cell to target processes therein. Furthermore, the mechanism by which such molecules would access their targets beyond the PV is not known. In the study presented here, we implemented the change mediated antigen technology (CMAT) to identify parasite molecules that are preferentially expressed inside infected cells. The approach was based on the reasoning that parasites express ‘new’ or antigenically modified molecules in the intracellular environment; therefore antiserum that is reactive to infected cells would contain immunoglobulins that are specific to these ‘new’ molecules. After adsorption of the antiserum with axenically cultured parasites, the antiserum was used to screen a parasite genomic expression library to identify genes encoding the molecules that are preferentially expressed in infected cells. We present for the first time evidence that some of these CMAT molecules accumulate in the PV and then traffic into the host cell in vesicles of distinct morphologies. Furthermore, several of these parasite molecules become localized in discrete compartments within the host cell.

## Introduction

Leishmaniasis is a disease that affects over 12 million people in approximately 88 countries. It manifests as varying types of cutaneous lesions, mucocutaneous lesions or visceral disease; the type of disease presentation is dependent on both the parasite species and characteristics of the host that are not completely defined. Parasite lesions are sites of inflammation where *Leishmania*-infected cells are exposed to a range of potential leishmanicidal activities. It is therefore no surprise that *Leishmania* infected cells exhibit altered responses including refractoriness to IFN-gamma activation [1], [2], inhibition of LPS induced signaling [3], altered calcium mobilization [4] and non responsiveness to inducers of cell death [5]. Together, these responses enable the parasite to persist within the host cell.

Studies on other intracellular microorganisms have shown that within the intracellular milieu, intracellular organisms elaborate molecules that target host cell functions. For example, in the apicomplexan organism *Toxoplasma gondii*, which resides in PVs that are mostly of parasite origin, molecules from secretory organelles have been shown to traffic beyond the pathogen vacuole and target activities in the host cell cytosol and nucleus [6]. Similarly, prokaryotic pathogens such as *Salmonella* and *Legionella* synthesize effector molecules in the intracellular milieu that subsequently gain access to the host cell cytosol via type III and type IV secretion apparatus, respectively [7], [8]. Importantly, these effector molecules are not expressed by organisms that are grown in liquid broth. Presuming that *Leishmania* species also elaborate molecules that target host cell processes, there is currently limited knowledge of the identity of such molecules and the conditions under which these molecules are synthesized and released into the infected cell. However, there is considerable evidence that *Leishmania* parasites also differentially express molecules in response to changes in their environment. For example, promastigote stage-specific molecules such as GP46, GP63 and lipophosphoglycan are rapidly turned off once parasites are internalized into macrophages or when promastigote forms are incubated in specialized media and growth conditions more suited for amastigote growth [9–1]. Also, LIT1 the ZIP family iron transporter was shown to be preferentially induced in parasites that reside within PVs only after several days of infection [12]. Following from these observations, it is plausible to expect that, as compared to axenically cultured organisms, parasites that grow within infected cells or inside infected hosts most likely elaborate new (or antigenically modified) molecules that permit them to function in the intracellular environment. It is to be expected though that the differential expression of such molecules would depend, in large part, on the actual details of parasite culture.

To identify parasite molecules that are preferentially expressed in infected cells, we elected to implement the change mediated antigen technology (CMAT), which is a variation of the in vivo induced antigen technology (IVIAT). Both of these screens are immunological screens that take advantage of the fact that antibodies can be raised to new molecules which are expressed by organisms that sense changes in their environment when they enter infected hosts as compared to organisms grown under standard laboratory culture [13], [14]. The immune serum that is elaborated in response to pathogens within infected hosts will include antibodies that are reactive to parasite structural molecules and housekeeping molecules that perform vital metabolic functions in the parasite. Incubation of this immune serum with axenic cultured organisms should result in strong reactivity of the antiserum with many of the latter parasite molecules. In the CMAT and IVIAT screens, immune serum is incubated with lysates of the pathogen grown under standard laboratory conditions to deplete the serum of antibodies that are reactive to parasite structural and housekeeping molecules. The depleted serum becomes enriched for immunoglobulins that are reactive to molecules preferentially expressed within the infected host. To date, CMAT and IVIAT have identified over 1000 *in vivo* induced antigens in infections including *Bacillus anthracis*, *Tannerella forsythia*, *Mycobacterium tuberculosis*, *Salmonella enterica* serovar Typhi, group A *Streptococcus*, *E. coli* O157:H7, *Vibrio vulnificus* and *V. cholera*, among others [15]–[21]. These newly identified antigens include new virulence factors in these organisms as well as pathogen proteins previously shown to be preferentially expressed in the infected host.

In the study presented here, serum reactive to infected cells was raised by immunization of mice with lysates of cells infected with *L. pifanoi* or *L. donovani* parasites. After adsorbing this serum extensively against axenic amastigote forms to remove antibodies that are reactive to molecules expressed by axenically cultured organisms, the adsorbed serum was used to screen inducible parasite genomic expression libraries. A few of the endogenous genes identified by the CMAT screen were analyzed for their time-course of expression and their distribution in infected cells. Evidence is presented that *Leishmania* parasite molecules identified by CMAT traffic outside the PV and are therefore putative candidate effectors.

## Materials and Methods

### Parasites and culture conditions

*Leishmania pifanoi* promastigotes (MHOM/VE/57/LL1) obtained from the ATCC were grown in Schneiders medium supplemented with 10% fetal calf serum and 10 µg/ml gentamicin at 23°C. *L. donovani* strain 1S-CL2D from Sudan, World Health Organization (WHO) designation: (MHOM/SD/62/1S-CL2D) was obtained from Dr. Debrabant (USDA, MD). Promastigotes of this parasite strain were grown in Medium-199 (with Hank's salts, Gibco Invitrogen Corp.) supplemented to a final concentration of 2 mM L-glutamine, 100 µM adenosine, 23 µM folic acid, 100 IU and 100 µg/ml each of penicillin G and streptomycin, respectively, 1×BME vitamin mix, 25 mM Hepes, and 10% (v/v) heat-inactivated (45 min at 56°C) fetal bovine serum, adjusted with 1 N HCl to pH 6.8 at 26°C. Generation of amastigotes forms was carried out as described [22]. *L. donovani* axenic amastigotes parasites were maintained in RPMI-1640/MES/pH 5.5 medium at 37°C in a humidified atmosphere containing 5–7% CO_2_ in air. *L. pifanoi* amastigotes were maintained in the amastigote medium above at 34°C. The RAW 264.7 murine macrophage cell line was cultured in RPMI supplemented with 10% fetal calf serum and 100 units Penicillin/Streptomycin at 37°C under a 5% CO_2_ atmosphere.

### Construction of inducible *Leishmania* genomic DNA expression libraries

Genomic DNA of *L. donovani* and *L. pifanoi* parasites was prepared using the TELT method [23]. The genomic DNA was digested with *Sau3*A and 0.5–2.0 kb fragments were size selected. BL21(DE3)-based inducible expression libraries were constructed with *L. pifanoi* and *L. donovani* fragments in pET30abc using techniques previously described [13], [24].

### Generation of antiserum and adsorbtion of serum to *Leishmania* amastigotes grown axenically

Macrophages infected for 24 H with amastigotes of *L. donovani* or *L. pifanoi* were lysed in lysis buffer (25 mM HEPES, 150 mM NaCl, 1% Triton X-100, 1x protease inhibitor pill (1 pill/10 mL), 20 ul/mL Phosphatase Inhibitor Cocktail 1, 20 ul/mL Phosphatase Inhibitor Cocktail 2 (Sigma), 50 uM NaVO_4_). 100 ug of lysate per mouse was emulsified in an equal volume of titermax gold adjuvant (Sigma Aldrich). BALB/c mice were immunized and boosted twice. Sera from 4 mice was combined and used in adsorptions. Antibodies reactive with antigens expressed by axenically grown parasites were removed by 4 successive incubations of sera with whole parasites (5×10^8^ parasites per adsorption) in PBS supplemented with 1% bovine serum albumin (BSA) and 0.05% sodium azide as described earlier [24]. Sera were further incubated with parasites lysed by French Press (4×10^9^ parasites) and heat treated parasites that had been charged onto microspheres (polystyrene microspheres, Bangs Labs, Fishers IN). An additional adsorption was performed with RAW 264.7 lysates charged onto microspheres. Aliquots were saved after each adsorption

### ELISA

Reactivity of adsorbed sera was determined by incubation of serially diluted sera in 96 well plates (Nalge Nunc International, Rochester, NY), that were pre-coated with lysates from axenic parasites, infected cells or uninfected cells. A Goat anti-mouse-HRP secondary antibody was applied. Plates were developed using HRP substrate solutions (BD Biosciences, San Jose, CA) and plates were read at 450 nm on a Powerwave 200 spectrophotometer (Bio-Tek, Winooski, VT).

### Primary screening of the protein expression library, identification of inserts

The genomic expression library was grown on LB medium containing kanamycin (50 mg ml^−1^) for 12–14 H at 37°C to generate plates containing approximately 200–500 colonies. Each plate was replicated using nitrocellulose disks that were then placed onto a LB plate containing kanamycin and IPTG (1 mM) and incubated for 5 H at 37°C to induce expression of cloned open reading frames (ORFs). The colonies were exposed to chloroform vapors for 20 min, then overlaid with nitrocellulose membranes. The membranes were incubated in PBS, pH 7.2, containing 0.5% Tween-20 (PBS-Tween) and 5% non-fat skim-milk. They were reacted with the adsorbed sera at a 1∶300 dilution in PBS-Tween for 1 H, then with peroxidase-conjugated goat anti-mouse immunoglobulin at a 1∶3000 dilution for 1 H. Reactive clones were detected by enhanced chemiluminescence (ECL kit, Amersham Pharmacia) and exposure to Hyperfilm (Amersham Pharmacia).

To confirm the immunoreactivity of the clones identified above, several colonies from the master plate in the apparent vicinity of the signal were streaked for single colony isolation onto LB plates. After overnight incubation, three single colonies isolated from each original colony were resuspended in fresh liquid LB medium containing kanamycin. Aliquots were blotted onto solid LB medium containing kanamycin and IPTG. Following the induction of recombinant protein expression, the blotted cells were lysed and re-screened using the same procedures as in the first round of screening. Two negative controls were included on each plate: pET30b/BL21(DE3) with no cloned insert, and a random clone that contained an insert but was non-reactive with sera.

### PCR amplification

PCR primers to amplify the gene transcripts of the antigens identified with CMAT were designed using the sequence in the clones or the sequence of the closest homolog available in the sequence database (www.genedb.org) as well as the 5′ splice leader. For example, for the IVI-59, the *L. infantum* ortholog (LinJ31_V3.1500) was used to design primers to amplify a transcript fragment corresponding to nucleotide positions +376 to +2113 of the *L. infantum* sequence. The PCR primers used were FB535 5′-CCATGGACCTGCCCACCGTCACATTC-3′ and FB536 5′-AGCTTGCCACGCTGCCACCTCTTTGAG-3′. The PCR product was electrophoresed in a 1% agarose gel and a fragment at the expected size location (1771 bp) was observed. The PCR fragment was excised from the gel, purified using a gel extraction kit (Qiagen) and cloned into a pET30 expression vector (Novagen) using restriction sites incorporated into the primers.

### Western Blot analysis

RAW 264.7 macrophages plated in 100 mm dishes were infected with *L. donovani or L. pifanoi* promastigotes. At various times, parasites were removed, cell monolayers washed twice with PBS and the infected macrophages were lysed directly in the plate with lysis buffer (25 mM HEPES, 150 mM NaCl, 1% Triton X-100, 1x protease inhibitor pill (1 pill/10 mL) (Roche Applied Science, Indianapolis, IN), 20 ul/mL Phosphatase Inhibitor Cocktail 1 (Sigma-Aldrich, St. Louis, MO), 20 ul/mL Phosphatase Inhibitor Cocktail 2 (Sigma), 50 uM NaVO_4_). Protein containing lysates were then cleared of cellular debris by centrifugation and protein concentration was determined using a Bradford Protein Assay (BioRad, Hercules, CA). SDS-PAGE was run on 50 µg of each sample on 12% polyacrylamide gels. Proteins were transferred to Immobilon P membrane (Millipore, Billerica, MA), the membranes were incubated with milk and then probed with primary antibody. After removal of primary antibodies and washing, membranes were incubated in the appropriate secondary antibodies conjugated to horse-radish peroxidase. Washed blots were incubated with chemiluminescence (ECL, Amersham) reagents. Antibody reactivity was visualized by exposure of blots to x-ray film. Some blots were stripped by incubation in 62.5 mM Tris HCl pH 6.8 supplemented with 20 mM 2-mercaptoethanol and 2% SDS, for 30 minutes at 50°C. The blots were then re-probed with other antibodies including the JLA20 the anti-actin antibody and E7 the anti tubulin antibody (both of these antibodies were obtained from the Developmental Studies Hybridoma Bank (University of Iowa).

### Immunofluorescence labeling and imaging

Infected cells on coverslips were fixed with 2% paraformaldehyde and processed as described previously [25]. Incubation with primary antibodies was performed in binding buffer supplemented with 0.05% saponin. Coverslips were incubated with the appropriate secondary antibodies into which the nucleic acid dye 4′,6-diamidino-2 phenylindole dihydrochloride (DAPI) had been added. Coverslips were mounted on glass slides with ProLong antifade (Molecular Probes). They were examined through a 63X oil immersion lens on a Zeiss axiovert 200 M integrated into a spinning disc confocal microscope technology from PerkinElmer (Waltham, MA) controlled by the volocity software. Z stack of optical sections spanning the entire cell were captured and then combined using the extended focus feature in the volocity software producing a 3D image.

### Immuno-electron microscopy

Two methods of cell preparation were used. In the first, cells infected for 72 H were fixed for 10 min at room temp then for 20 minutes at 4°C in a mixture of 4% formaldehyde and 1% glutaraldehyde in 0.1 M cacodylate buffer (pH 7.2). The pellet, embedded in low-gelling-point agarose to facilitate handling, was dehydrated in an ethanol series, then embedded in LR White resin (Electron Microscopy Science, Fort Washington, PA) and polymerized at 50°C. Sections were cut with a diamond knife on an RMC MT-6000-XL ultramicrotome and collected on Formvar-coated nickel grids. In the second method, 72 H-infected cultures were scrapped into a small volume of 0.15 M sucrose in RPMI. The cells were pelleted in 1.5 ml vials and they were rapidly frozen in a Baltec HPM 010 high-pressure freezer (Boeckeler Instruments, Tucson, AZ). Freeze-substitution was carried out 5 days at −80°C in anhydrous acetone containing 2% OsO_4_/0.5% uranyl acetate. The temperature of the freeze-substitution medium was then increased from −90°C to −60°C over 12 H, and the samples were washed with anhydrous acetone three times at −60°C. Lowicryl HM20 (Electron Microscopy Sciences) resin embedding was performed by a stepwise increase in resin concentration from 0, 33, 66, to 100% over 48 H at −60°C. The samples were then washed three times with 100% resin and polymerized under UV light for 24 H at −60°C. Once completely polymerized, the samples were slowly warmed from −60 to 20°C over a 4 H period as described in [26]. Thick sections (80 to approx. 120 nm thick) of Lowicryl HM20 embedded cells were mounted on gold slot grids coated with formvar (Electron Microscopy Sciences, Hatfield, PA). Grids were blocked with 1% dry milk in phosphate-buffered saline (PBS, pH 7.2), treated overnight at 4°C with primary antibody or normal mouse serum (control) diluted 1∶10 in PBS (chemically fixed samples) or 1∶25 (cryofixed samples), washed with high salt Tris/Tween buffer (0.5 M NaCl; 0.02 M Tris, pH 8.0; 0.1% Tween-20) and reacted for 1 H with secondary antibody conjugated to 12 nm or 18 nm gold (goat anti-mouse IgG; Jackson ImmunoResearch Laboratories, West Grove, Pa.) diluted 1∶30 in PBS. In experiments with antisera to cTXNPx (Rat) a secondary antibody conjugated to 5 nm gold was used. Some sections were exposed to gold-labeled secondary antibody alone as an additional control. After washing, grids were floated on Trump's fixative for 10 min and washed with deionized water. Sections were then post-stained with 0.5% uranyl acetate and lead citrate and were examined with a Zeiss EM-10CA transmission electron microscope

### Gold count

Immuno-EM labeling was performed as described. Images were captured on Hitachi 700 TEM. Images were opened in ImageJ. The area of the parasite, PV lumen, cytosol and nucleus were measured and normalized using the measure bar on the image. Gold particles were counted in each compartment and normalized by dividing by the area of the compartment (µm^2^). N = 10.

### Nuclear fractionation

RAW264.7 cells were cultured as described and infected with *L. pifanoi* at ratio of 1∶10. Cells were harvested at 48 and 72 H. Cells were washed with cold PBS three times. They were then lysed in a nuclear extraction buffer (10 mM Hepes, 10 mM KCl, 1% Triton X 100, 0.1 mM Na_3_VO_4_, 2 mM MgCl_2_, 0.5 mM DTT, 1 mM PMSF) and laid over 20% Ficoll-Paque (Pharmacia) as described in [27]. The lysate was centrifuged at 13,000 rpm to separate the cytoplasm from the nuclei. The supernatant was removed and saved as crude cytosolic fraction. The nuclear pellet was washed 3 times using the nuclear extraction buffer. Aliquots of this fraction that were monitored by light microscopy did not reveal the presence of intact parasites. The nuclear pellet was then lysed in RIPA buffer to release nuclear proteins. The Protein containing lysates were then cleared of cellular debris by centrifugation and protein concentration was determined using a Bradford Protein Assay (BioRad, Hercules, CA). SDS-PAGE was run on 50 µg of each sample on 12% polyacrylamide gels. Proteins were transferred to Immobilon P membrane (Millipore, Billerica, MA).

## Results and Discussion

### Generation of antiserum preferentially reactive to *Leishmania*-infected cells

We elected to focus our studies on two *Leishmania* species: *L. mexicana pifanoi* (MHOM/VE/57/LL1) a member of the *L. mexicana* complex and *L. donovani* (MHOM/SD/62/1S-CL2). The rationale for selecting these parasites included the following: 1) Experimental conditions for the transformation of these parasites lines from the promastigote form to the amastigote form and their maintenance as amastigotes is well established [22], [28]–[34]. Obtaining sufficiently adsorbed antibodies is a critical step in the CMAT method, so it was important to have parasite lines with stable growth in the amastigote stage under reproducible conditions. 2) These two parasite species reside in morphologically distinct PVs. *L. pifanoi* parasites reside in communal PVs that undergo progressive distention over the length of the infection period. In contrast, *L. donovani* parasites reside in tight PVs from where daughter progeny segregate into new PVs. Since we did not know which of these two intracellular environments would represent a greater change in the parasite's environment that would induce differential gene expression, it was prudent to use both parasites in our analyses.

**Figure 1:**
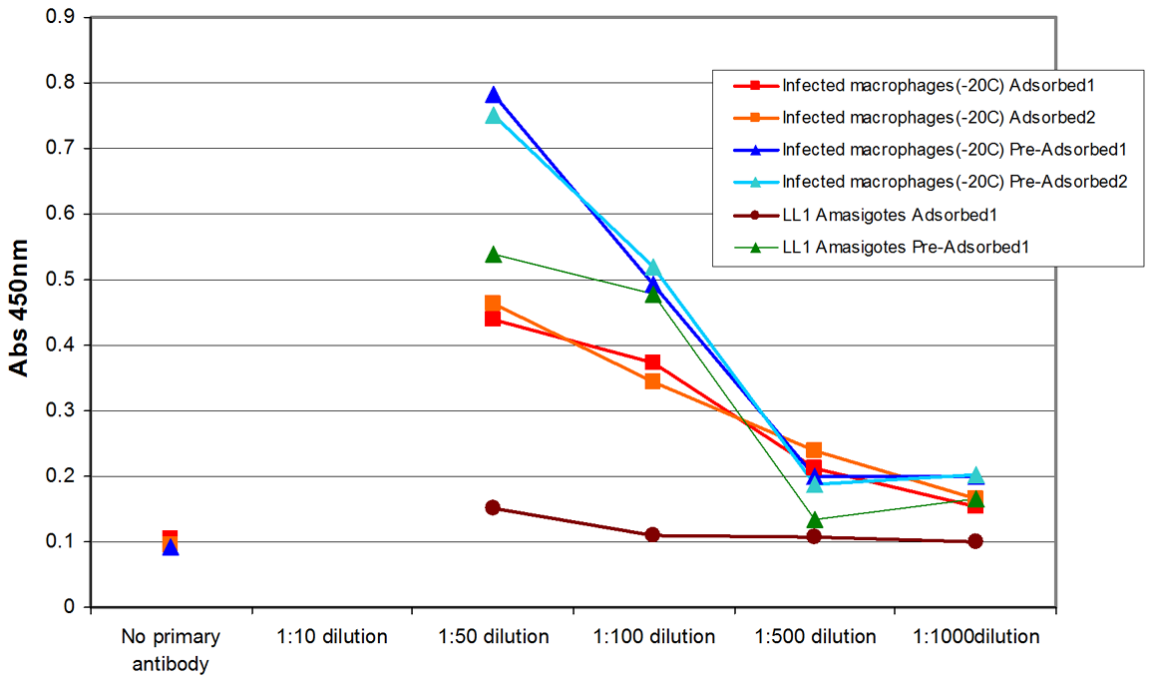
Adsorbed sera lose reactivity to axenically cultured parasites. Ninety six well plates were coated with lysates from axenically cultured amastigotes (LL1 amastigotes) or lysates from macrophages infected with amastigotes for 24 H (infected macrophages −20C). ELISAs were performed with two batches of sera before (Pre-adsorbed) and after (Adsorbed) adsorption. The optimal lysate concentrations used to coat the plates as well as sera dilutions were first established. This figure is representative of several experiments to evaluate the reactivity of the absorbed serum. Each data point is the average absorbance from triplicate wells.

To obtain serum that was preferentially reactive to infected cells, lysates of RAW 264.7 macrophages infected for 24 H with axenic amastigote forms of either *Leishmania* species were prepared and used to immunize BALB/c mice. Immune serum was collected and adsorbed extensively with whole amastigotes, amastigote lysates, heat-killed amastigotes, and lysates of RAW 264.7 cells as described in the Materials and Methods section. The resulting adsorbed serum had minimal reactivity with lysates from axenic parasites, but retained reactivity to lysates from infected cells. A representative ELISA showing the reactivity of pre-adsorbed and adsorbed sera to axenic *L. pifanoi* (LL1) lysates and infected cell lysate is shown in Figure 1. Even at a 1∶50 dilution of the adsorbed antisera, these sera had no reactivity to lysates prepared from axenic parasites even though they retained their reactivity to lysates prepared from infected cells. Similar results were obtained with *L. donovani* reactive serum (not shown). This implied that antibodies reactive to molecules expressed by axenic parasites were depleted during the adsorption protocol. However, the possibility that the adsorption of molecules expressed at low levels by axenic parasites was incomplete cannot be ruled out. The CMAT is an immunoscreen and its success is dependent on the quality of the adsorbed immune serum as judged by its titer and the diversity of the antigenic determinants to which the serum is reactive. In the future, sera with more diverse immune reactivity might be obtained by immunizing outbred mice species or rabbits. Alternatively, pooled convalescence sera from human subjects would provide a rich serum source with diverse antigenic reactivity.

### Isolation and identification of *Leishmania* molecules

The adsorbed immune sera were used to identify the new antigenic parasite molecules that they were reactive to within infected cells. To achieve this, these sera were used to screen inducible expression libraries in *E. coli* BL21(DE3) cells containing *Leishmania* genomic inserts from 0.5 to 2.0 Kbp cloned in pET30abc (Novagen). The use of genomic expression libraries is feasible in leishmaniasis because only a few parasite genes have been shown to have introns. After a primary screen of 20,000 clones from the *L. pifanoi* library and 20,000 clones from the *L. donovani* library, we obtained 27 clones reactive to the *L. pifanoi* serum and 30 clones reactive to the *L. donovani* serum. Although these library screens did not represent a total coverage of the genome, we decided to proceed in order to evaluate the characteristics of the molecules that were being identified by this approach. Twenty total clones remained robustly positive after tertiary screens. Four clones reactive to *L. pifanoi* and seven reactive to *L. donovani* were randomly selected for further analyses. The inserts in the clones were sequenced and the sequences were used to BLAST (Basic Local Alignment Search Tool) search *Leishmania* genomes in the sequence database www.genedb.org. Table 1 shows the most likely homologs of the *in vivo* induced genes (IVI) identified by CMAT. Six of the 10 IVI genes selected were homologs of genes that encode hypothetical proteins for which the *Leishmania* genes have no known homologs in organisms other than kinetoplastids. Three of the remaining genes belonged to gene families that encoded parasite molecules that had previously been shown to be preferentially expressed in *Leishmania* amastigote forms. The tryparedoxin peroxidase gene had been characterized in promastigote and amastigote forms. There was only one instance (IVI-7.1), in which we were not able to associate a gene from the sequenced genomes to the sequence in the CMAT clone even though a homologous sequence was found in the *L. infantum* genome.

**Table 1:** *Leishmania* effector molecules identified by CMAT.

Clone name	Homologous Gene Indentified	Chromosome	Signal sequence	Comment
IVI- 4	LmjF25.0450 LinJ25.0460	25	No	Hypothetical protein, possibly GTP binding protein
IVI-14	LmjF04.1020	4		Hypothetical protein
IVI-16	LmjF15.1060 LmjF15.1140 LmjF15.1100	15	No	Tryparedoxin peroxidase/peridoxins
IVI-18	LmjF30.2390	30	No	Hypothetical protein serine peptidase with hemopexin repeats
IVI- 3.1	LinJ08_V3.0400 LmjF08.0390	8	No	Hypothetical protein
IVI-7.1	no predicted protein			Sequence present in genome
IVI-7.2	LmjF10.1225	10		Hypothetical protein
IVI-59	LinJ31_V3.1500 LmjF31.1470	31	Yes	Hypothetical protein
IVI-62	LmjF14.1440 LinJ07_V3.0470	14	No	Cysteine proteinase
IVI-63	LinJ30_V3.1480 LmjF30.1420	30	No	Ama 1
IVI-64	LmjF34.1080 LinJ34_V3.1150	34	No	amastin-like surface protein

A concern with the selection of the parasite lines used in this study was that genes in organisms such as *L. pifanoi* and *L. donovani*, whose genomes had not been completely sequenced, might not have homologs in the genomes that had been sequenced. This concern was somewhat mitigated by the study in which comparison of a few selected open reading frames (ORFs) of *L. mexicana* were found to have a 89.5% identity to *L. major* ORFs [35]. However, a study by Peacock and colleagues [36], in which the genomes of *L. braziliensis*, *L. infantum* and *L. major* were compared, identified 78 genes that are restricted to either genome and observed an additional 200 sequence differences. Whether any of such unique genes would have been detected in our screen is unknown. With the exception of IVI-7.1 noted above, all the IVI genes identified from the *L. pifanoi* and *L. donovani* expression libraries were found to have homologs in the *L. major*, *L. infantum* and *L. braziliensis* sequenced genomes. The sequence of the *L. mexicana* and *L. donovani* genomes are now available.

### Molecules identified by the CMAT screen

**Figure 2:**
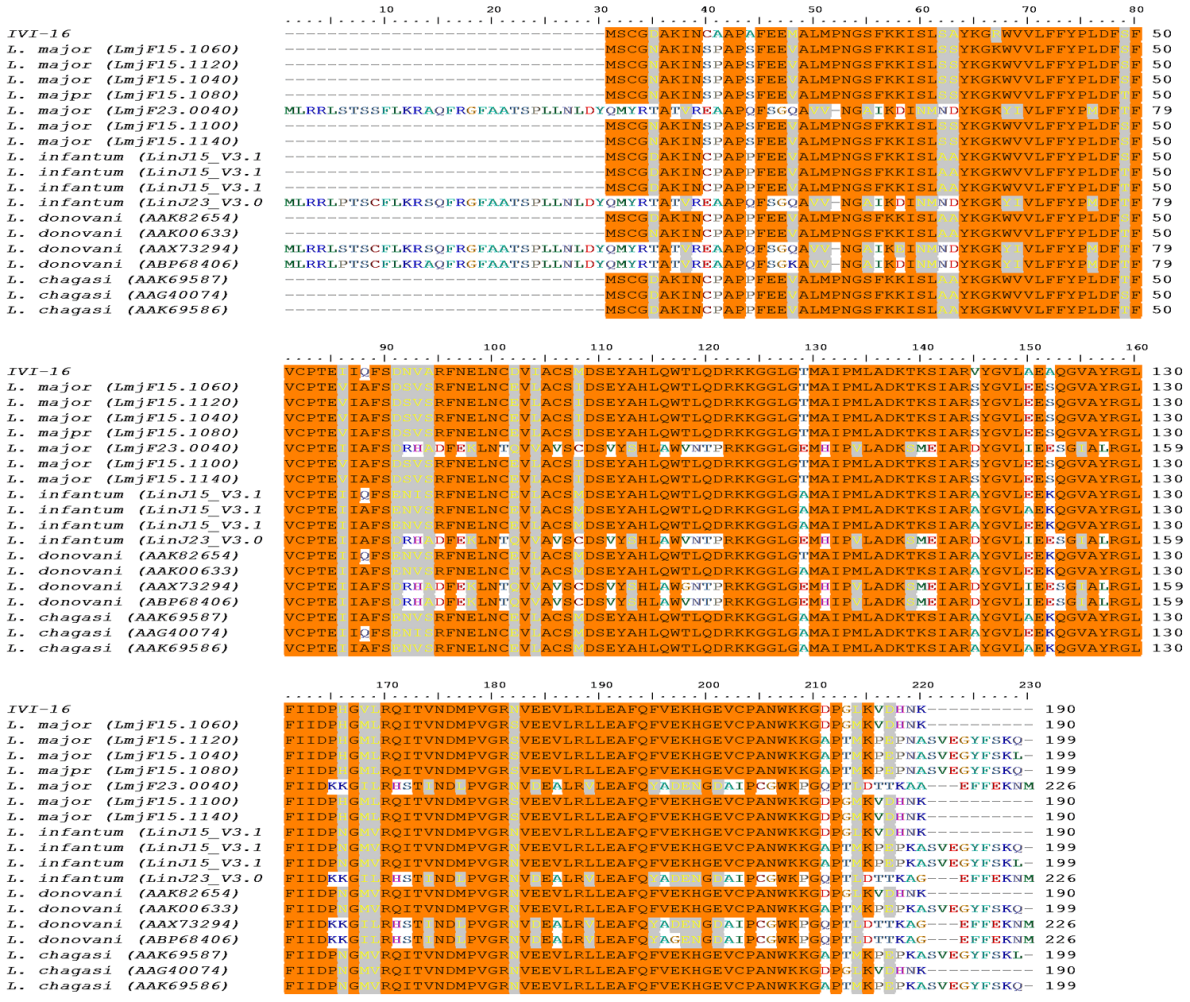
Sequence comparison of IVI-16 with TXNPx gene sequences. The *L. pifanoi* IVI-16/TXNPx full length gene obtained by amplification of cDNA from infected cells, was aligned with TXNPx genes from the GeneDB genome database using ClustalW.

*An intracellular variant of Typaredoxin peroxidase*. The BLAST search of the sequenced genomes with the IVI-16 sequence revealed that this CMAT clone contained a *L. pifanoi* homolog of tryparedoxin peroxidases (TXNPx). The clone contained the 3′ region of the TXNPx gene and a segment of the contiguous gene on the chromosome annotated as the developmentally regulated gene (DRG). Induction of IVI-16 expression with IPTG produced a 15 kDa recombinant protein that included the c-terminus of the TXNPx gene and vector sequences. A stop codon between the TXNPx and DRG sequences prevented expression of the DRG sequence in the clone. Using primers designed from the sequence in the IVI-16 clone and the *Leishmania* splice leader, we obtained the entire coding sequence of the gene in the CMAT clone by amplification of cDNA prepared from *L.pifanoi*-infected cells. A phylogentic analysis of the relatedness of this sequence with other available TXNPx sequences showed that this gene is most closely related to AAX47428, a gene from *L. amazonensis*, which like *L. pifanoi* is a member of the *L. mexicana* complex (Figure S1). An alignment of the predicted protein sequence of the new IVI-16 gene product with the available sequences of TXNPx in the GeneDB genome database is shown in Figure 2. In addition to amino acid sequence differences in the C-terminus of these molecules, some TXNPx molecules including the IVI-16 gene cloned here are nine amino acids shorter than other TXNPx sequences. This is consistent with a previous study that demonstrated the amastigote variant (P1) of *L. chagasi* peroxidoxin is shorter by 9 amino acids than two other variants (P2 and P3) preferentially expressed in *L. chagasi* promastigotes [37]. This suggested that the gene identified by CMAT is amastigote specific. This cloned variant of the *L. pifanoi* TXNPx gene is hereto forth referred to as IVI-16/TXNPx. We also noted that there are additional amino acid differences throughout the length of the protein that might result in antigenic differences between this protein and other TXNPx expressed by *Leishmania* parasites.

**Figure 3:**
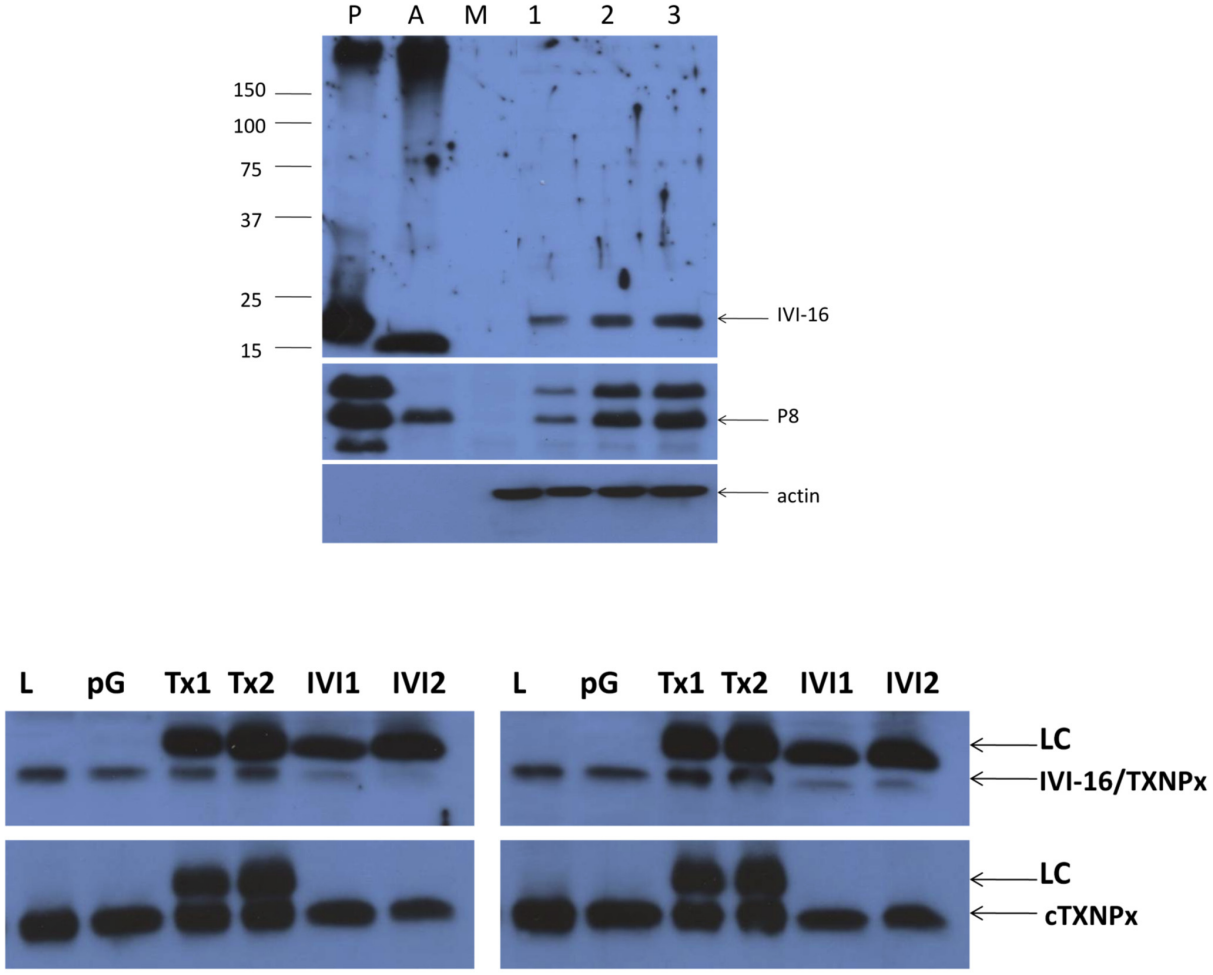
Western blot analysis of the expression of the endogenous gene reactive with the antiserum to IVI-16/TXNPx. A. Lysates were obtained from promastigotes cultures (P), axenic amastigotes (A), uninfected macrophages (M) and macrophages infected with promastigotes for 24, 48 or 72 H. These were run on 12% PAGE and blotted onto nylon membranes. The membrane was probed with antiserum raised to the IVI-16/TXNPx recombinant protein. The blot was stripped and probed with antibody to P8, which is a complex expressed by stationary stage promastigotes, axenic amastigotes and infected cells. The blots were stripped again and probed with culture supernatant produced by the JLA20 hybridoma line. B. Western blot of lysates from depletion experiments using IVI-16/TXNPx antiserum. Lysates from 48 and 72 H infections were incubating with 2 (IVI 1) and 4 (IVI 2) µl of IVI-16/TXNPx antiserum or 2 (Tx1) or 4 (Tx2) ul of antiserum to cTXNPx (rat antibody). Protein G was added to deplete all molecules reactive with the antisera. The remaining supernatant fluid was run on 15% PAGE and blotted. The blot was probed with the IVI-16/TXNPx antiserum, stripped and re-probed with cTXNPx antiserum. This experiment was performed twice. [LC  =  light chain].

Tryparedoxin peroxidases (also called peroxidoxins or peroxiredoxins) belong to a gene family that has been characterized extensively in *Leishmania* [37], [38], [39] and in other organisms as well [40]. These molecules are antioxidants that detoxify peroxides, hydroxyl radicals and peroxynitrite by several mechanisms including oxidoreduction. These molecules are vital to the parasite since it is believed that both in the insect vector and in the mammalian host *Leishmania* encounter a range of toxic oxidative environments that it must harness to survive. Moreover, recent reports have implicated TXNPx in parasite dissemination and in light of the inability to generate parasites that lack this gene, it was suggested that TXNPx are necessary for parasite survival [38]. TXNPx variants have been described that localize to discrete cellular compartments such as the mitochondrion and the cytosol within the parasite [37], [38]. As discussed above, the CMAT screen resulted in the cloning of the *L. pifanoi* IVI-16/TXNPx. Given the relatedness of the cloned gene to other TXNPx genes, it was intriguing that antibodies to this molecule were not adsorbed out after incubations with axenic parasite lysates. The possibility that the adsorption protocol implemented was incomplete could not be ruled out; alternatively, it was likely that the IVI-16/TXNPx molecule identified is a variant that is antigenically different from other TXNPx variants expressed in the parasite. Several pieces of evidence support the latter statement. Western blot analysis of lysates obtained from cultured organisms, as well as lysates from infected cells and uninfected macrophages, probed with antiserum raised with the IVI-16/TXNPx recombinant protein showed that the antiserum was not reactive to lysates of uninfected macrophages (lane M) but exhibited strong and specific reactivity to a molecule of ∼20 kDa in lysates obtained from macrophages infected for 24, 48 or 72 H with *L. pifanoi* promastigotes (Figure 3). Promastigote stage parasites transform into the amastigote form in 10–24 H after entry into macrophages [41]. No comparable reactivity was observed to lysates of cultured promastigotes alone (P) or axenic amastigotes (A). The same lysates were tested for their reactivity to P8 antiserum. P8 is a molecular complex that is expressed in stationary stage promastigotes and axenic amastigotes of *L. pifanoi* [42]. The complex was indeed identified within the parasite lysates as well as within infected cells (Figure 3). The anti-P8 antiserum was also non-reactive with lysates from uninfected macrophages and actin levels in lysates from uninfected and infected macrophages confirmed that the gel was loaded equally (Figure 3). Northern blots probed with the IV1-16/TXNPx sequence under stringent conditions revealed the presence of transcripts of the endogenous IV1-16/TXNPx gene in axenic promastigotes and amastigotes as well as infected cells, which is consistent with reports of *Leishmania* genes being constitutively transcribed (not shown).

Additional evidence that the IV1-16/TXNPx molecule identified by the CMAT screen is antigenically distinct from a cytosolic variant of TXNPx (cTXNPx) expressed by these parasites was obtained in protein depletion experiments. Here, lysates obtained from infected macrophages were incubated with the anti-IVI-16/TXNPx antiserum. The endogenous molecule in the lysate that is reactive to this antiserum was depleted by adding protein G to the lysate. The supernatant fluid from this depletion experiment was then analyzed in Western blot assays for reactivity to the anti-IVI-16/TXNPx antiserum and to an antiserum that is reactive to the cTXNPx [39]. The incubation of infected cell lysates with the anti-IVI-16/TXNPx depleted the endogenous molecules recognized by that antiserum while the molecule that is recognized by the antiserum to cTXNPx was not depleted (Figure 3B). Taken together, these results show that IV1-16/TXNPx molecule, which is expressed within infected cells, is antigenically different from the cTXNPx that is also expressed in infected cells as well as by axenically cultured organisms.

### Other CMAT molecules

Six of the CMAT clones were homologs of genes annotated as encoding hypothetical proteins in the sequenced genomes (Table 1). This proportion of hypothetical proteins identified by the CMAT screen should be expected since only a third of the genes in the *Leishmania* genome have known homologs [43]. Application of sequence analyses tools on the homologs of the genes that were identified revealed that two of these genes have domains that might be suggestive of their function. The LmjF25.0450 homolog identified by IVI-4 was found to contain a GTP-binding domain, which suggests that this molecule might play a role in signal transduction [44]. An NCBI PSI-blast of the LmjF30.2390 gene identified by IVI-18 revealed that in addition to its relatedness to serine peptidases, it contains both a leucine rich repeat region (LRR) and hemopexin repeats. Over 100 proteins in *L. major* have been estimated to contain LRR sequences, which mediate protein-protein interactions. Another member of the LRR superfamily, LinJ34.0570, was recently described and implicated in antimony resistance [45]. Hemopexin repeats in proteins have been shown to mediate interactions with heme [46]. This is of relevance to *Leishmania* biology since it has been shown previously that *Leishmania* infections modulate heme degradation in infected cells, which controls the capacity of the infected cell to elaborate responses such as superoxide production that are dependent on heme availability [25]. It is also of interest that a molecule with heme binding activity was recently described on the surface of *L. infantum* amastigotes [47]. Future studies on these molecules should be illuminating.

**Figure 4:**
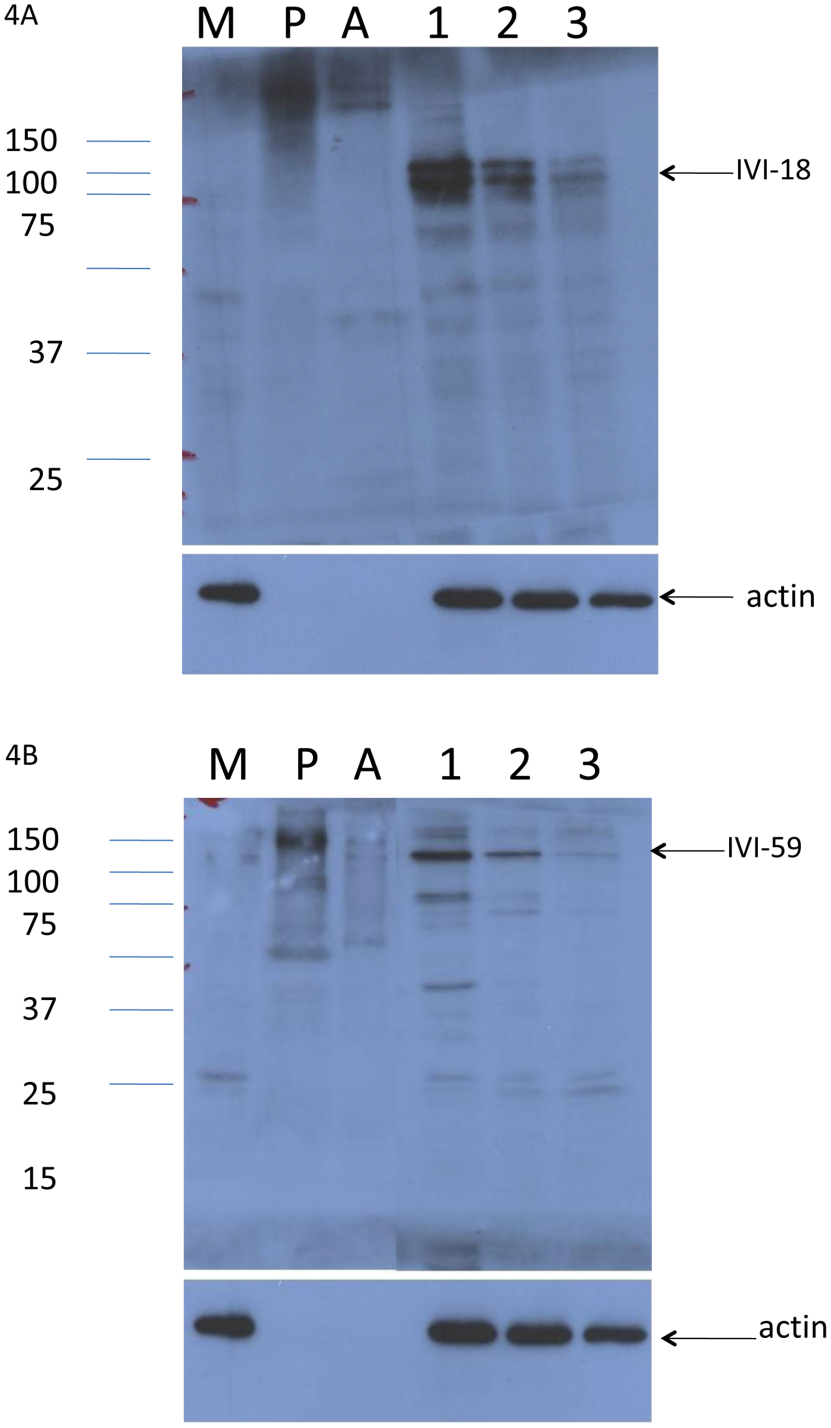
Western blot analyses of the expression of the endogenous genes reactive to IVI-18 and IVI-59 antisera. Lysates obtained from promastigotes cultures (P), axenic amastigotes (A), uninfected macrophages (M) and macrophage cultures infected for 24, 48 or 72 H were run on 12% PAGE and blotted onto nylon membranes. A. A membrane was probed with the antiserum that was raised to IVI-18 recombinant fragment. B. Another membrane was probed with antiserum that was raised to a recombinant fragment of IVI-59. The membranes were stripped and probed with the anti-actin JLA20 monoclonal antibody. This experiment is representative of at least 4 similar experiments.

Antisera were generated to the recombinant molecules encoded by the original library clones in pET30. The antisera were first confirmed for their reactivity to the recombinant antigens (not shown) before they were used to detect the endogenous molecules expressed in infected cells by Western blot assays, in immunofluorescence assays and immune-electron microscopy. Only antisera raised to the recombinant products of IVI-4, IVI-18 and IVI-59, which are the homologs of LmjF25.0450, LmjF30.2390 and LinJ31_V3.1500 respectively, reacted robustly to cells infected for 24 to 72 H. These antisera were reactive to infections with either the *L. pifanoi* or *L. donovani* parasite lines used to initiate the studies. Consequently, only analyses performed on *L. pifanoi* infected cells are presented. The antiserum to IVI-4 was poorly reactive in Western blot assays but exhibited specific reactivity to a parasite structure within infected cells (see below). Representative Western blot analyses to evaluate the expression of the endogenous genes identified by IVI-18 and IVI-59 are shown in Figure 4. In Figure 4A the reactivity of antiserum to IVI-18 is shown. This antiserum was not reactive to lysates of uninfected macrophages (lane M). In lysates prepared from macrophages infected for 24 to 72 H, a doublet of ∼95 kDa is recognized by this antiserum. In contrast, no molecule of comparable molecular weight is recognized in lysates prepared from stationary promastigote cultures or lysates from axenic amastigotes. In Figure 4B, the reactivity of the antiserum to IVI-59 is shown. This antiserum too was not reactive to lysates from uninfected macrophages (lane M). The IVI-59 antiserum was reactive to a molecule in infected cells of ∼125 kDa. Other bands in infected samples were most likely the result of protein degradation since the intensity of these bands varied with each lysate preparation. This antiserum had limited and inconsistent reactivity to lysates from axenic amastigotes and lysates obtained from stationary stage promastigotes. Moreover, any reactivity that was observed to promastigote or amastigote preparations was at a higher molecular weight. Together these results show that the *L. pifanoi* homolog of LmjF30.2390 and LinJ31_V3.1500 are preferentially expressed within infected cells. The apparent reduction in the levels of the endogenous proteins at the 72 H point was most likely due to the loss of infected cells that detach easily from the culture dish.

The three remaining molecules that were identified by the library screens were found to be homologs of previously annotated genes in the sequenced genomes. IVI-64 had similarity to amastins, which are a family of proteins that have been shown to be differentially expressed in *Leishmania* parasites [48], [49]. Alignment of the IVI- 64 sequence with sequences of amastin genes showed that it had highest similarity to LmjF34.1080 (Figure S2), which is an amastin variant predicted to be expressed on the parasite surface of amastigotes forms [46]. Knowledge of the conditions that result in the differential expression of individual members of the amastin gene family is incomplete. However, it has been shown that amastins gene expression, analyzed at the mRNA level, is regulated by the pH of their environment and that the highest accumulation of amastins is evident after 5–7 days of amastigote culture [48], [49]. It is likely that similar to the situation with IVI-16/TXNPx, the immunization with lysates from infected cells resulted in the generation of antisera to an antigenically distinct variant of amastins that is preferentially expressed within infected cells.

The IVI-63 had similarity to the *am3* gene (encoding for Ama1 protein), which was previously identified from a differentially expressed cDNA library and shown to be amastigote specific [50]. Although the time course of appearance of the endogenous protein was not evaluated in that study, transgenic parasites with an episomal copy of the gene, were shown to express this protein on the surface of parasites within infected cells. Further observations on this molecule are described below. Finally, BLAST of the NCBI database with the IVI-62 sequence showed that it had 99% similarity to the *L. infantum* cysteine proteinase b gene (AJ420286). Alignments of the IVI-62 sequence with the sequence of other cysteine proteinases (not shown) have provided additional evidence that it is indeed a cysteine proteinase.

### Localization of endogenous IVI molecules in infected cells

**Figure 5:**
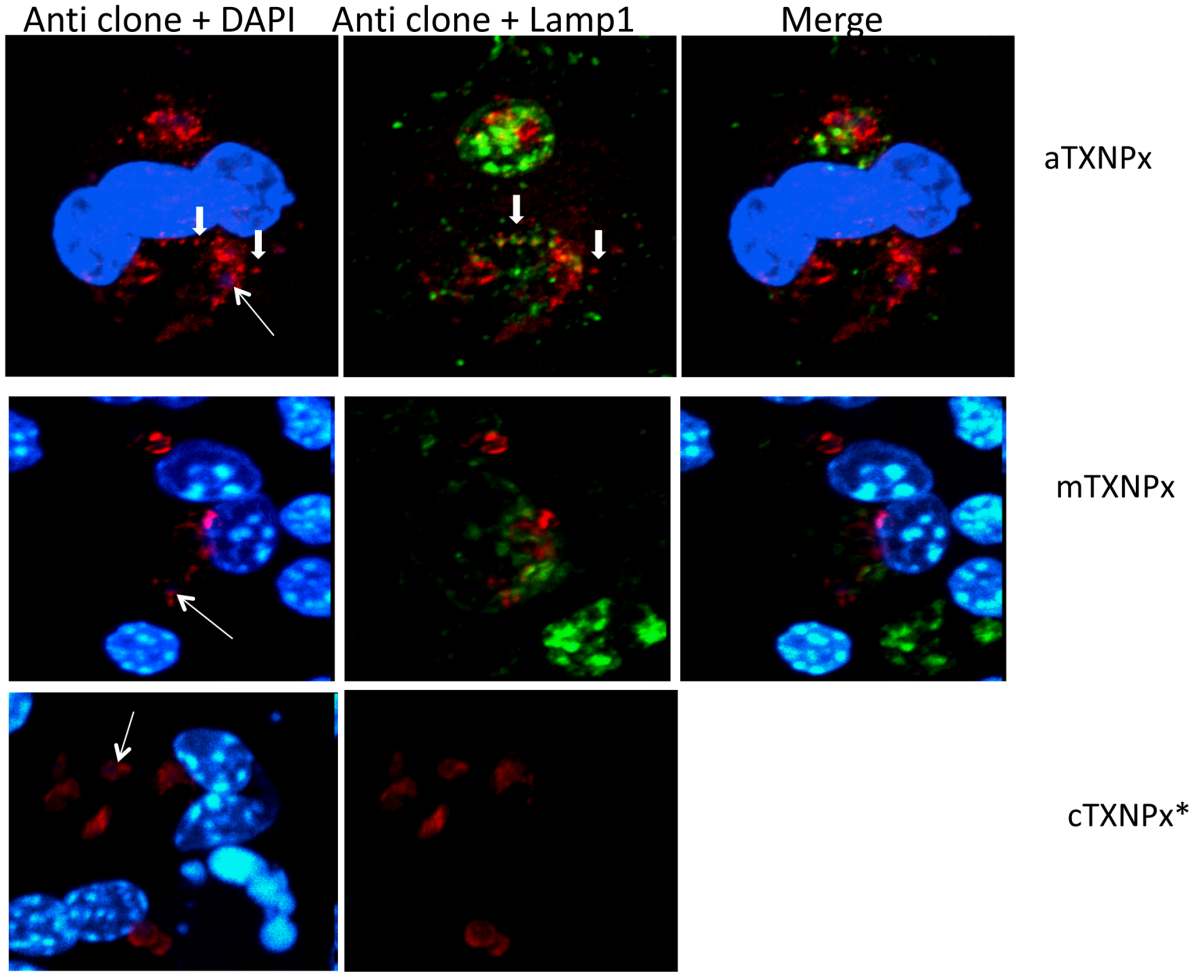
Immunolocalization of the endogenous IVI-16/TXNPx molecule, reactive with IVI-16/TXNPx antiserum. RAW 264.7 macrophages on coverslips were infected for 72 H and then fixed. Immunofluorescence staining was performed with antisera raised to the IVI-16/TXNPx recombinant, and antisera to the mitochondrial TXNPx (mTXNPx) and cytoplasmic TXNPx (cTXNPx). LAMP-1(green label) reactivity identifies lysosomes and delineates the PV membrane. DAPI (blue) labels nuclei. Specific reactivity by each antiserum is in red. The thin white arrows point to representative parasites in PVs. The thick arrows points to regions of antibody reactivity outside the parasite. Covers slips that were incubated with anti-cTXNPx were not assessed for LAMP-1 reactivity (re- both are rat antibodies). Secondary antibodies alone produced background reactivity. These images captured with a Zeiss axiovert 200 M integrated into a spinning disc confocal microscope technology from PerkinElmer (Waltham, MA) controlled by the volocity software. Z stack of optical sections spanning the entire cell were captured and then combined using the extended focus feature in the volocity software producing a 3D image. Immunofluorescence assays are representative of more than 3 experiments with these antisera.

Immunofluorescence assays and immuno-electron microscopic analyses were performed to determine the distribution within infected cells of the endogenous molecules that were identified by the CMAT screen. The distribution of the endogenous IVI-16/TXNPx molecule within infected cells was determined. The IVI-16/TXNPx antiserum labeled the parasite within infected cells (Figure 5, [thin white arrow points to a representative parasite]). There was also labeling of the PV in regions where there were no parasites. In addition, the antiserum labeled structures in the cytosol outside the PV (thick white arrows). The pattern of labeling of endogenous IVI-16/TXNPx was compared to the pattern of labeling of previously described antisera to the cTXNPx [39] and the mitochondrial TXNPx (mTXNPx) [38]. Both the antiserum to cTXNPx and mTXNPx exhibited specific and restricted labeling of the parasite within infected cells with patterns that were consistent with cytoplasmic staining and mitochondrial staining respectively; they did not label any structures outside the parasite.

**Figure 6:**
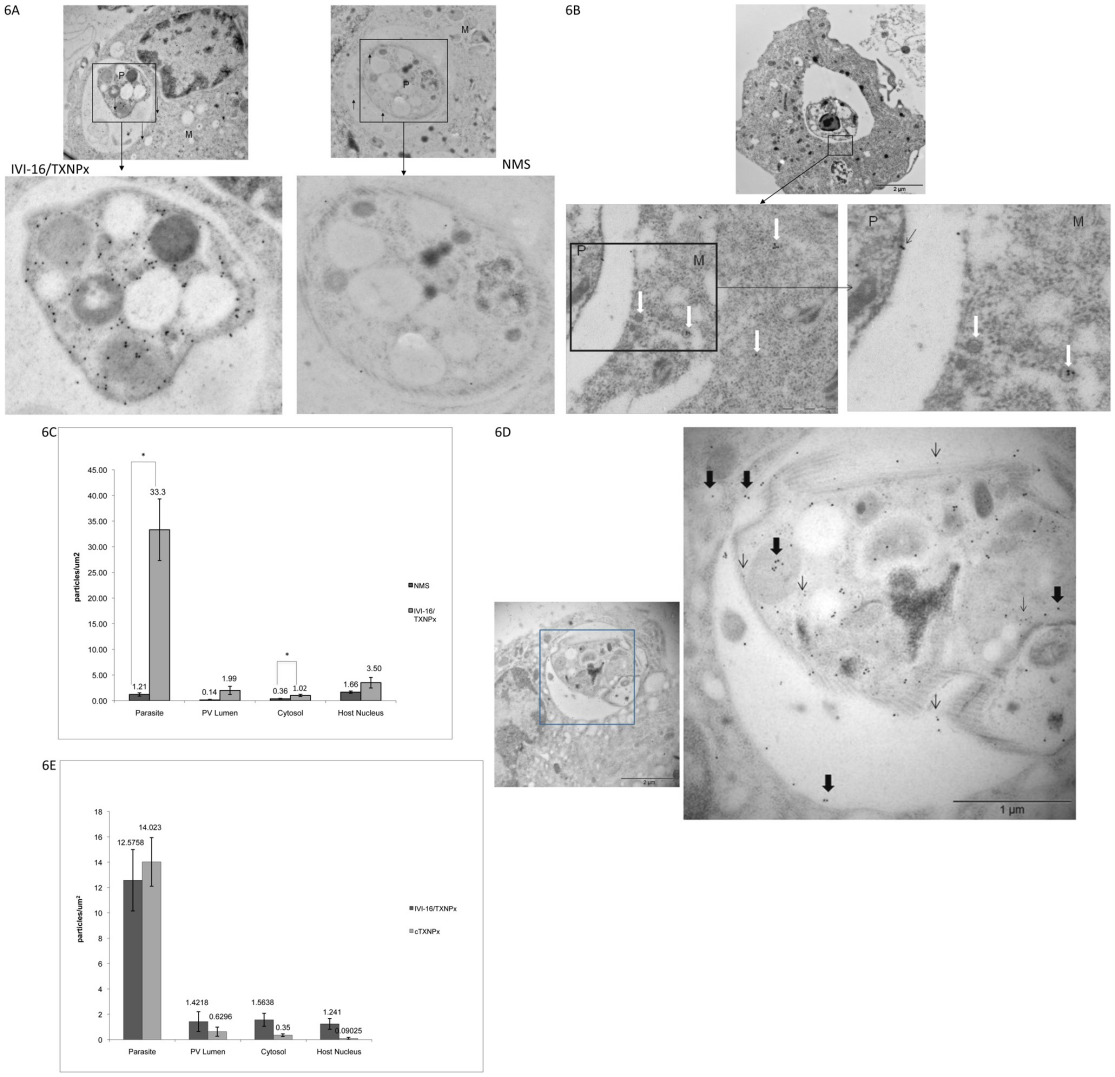
Localization of the endogenous molecule reactive with IVI-16/TXNPx antiserum by Immuno-EM. A. Cryofixed samples embedded in Lowicryl HM20 and sectioned were incubated with antiserum to IVI-16/TXNPx. This was followed by incubation with an anti-mouse 18 nm-gold conjugated antibody. Consecutive sections were incubated with normal mouse serum (NMS). Arrows in Figure show gold particles. B. Gold particles on sections incubated with either the antiserum to IVI-16/TXNPx or NMS were enumerated and the data was plotted. At least 10 cells were counted per experiment. The difference between counts from the specific antiserum and NMS were assessed in a t-test. Asterix denote significant difference, P<0.5. C. Infected cells were chemically fixed then embedded in LR White resin. Sections mounted on nickel grids were incubated with the antiserum to IVI-16/TXNPx followed by incubation with an anti-mouse 15 nm-gold conjugated antibody. Thin black arrow points to gold particles on the parasite; bold white arrows point to gold particles in the host cell. D. Double labeling experiment; samples from cryofixation were incubated with antiserum to IVI-16/TXNPx (mouse) as well as the antiserum to cTXNPx (rat). The reactivity of cTXNPx was monitored with an anti-rat 5 nm gold conjugated secondary antibody. Thin black arrows point to examples of 5 nm gold; thick black arrows point to examples of 18 nm gold. E. Gold particles in double labeling experiments were enumerated and plotted. Asterix denote significant difference, P<0.05. Legend: M = macrophage; P = Parasite; N = Nucleus.

To obtain greater resolution of the structures that were reactive to the IVI-16/TXNPx antiserum, immuno-labeling of infected cell sections on grids that were processed for EM analysis was performed. Two protocols for sample preparation for EM analyses were used. LR white embedding of chemically fixed cells permitted better resolution of membranous structures as compared to cryofixation of samples. However, all the antisera that were used in this study labeled sections prepared with the latter protocol with greater sensitivity. EM images using both of these protocols are presented in Figure 6. In Figure 6A a representative example of the pattern of labeling observed when the IVI-16/TXNPx antiserum was applied on samples obtained by cryofixation is shown. In these samples the antiserum labeled the parasite, the PV lumen and the cell cytosol outside the PV (black arrows). The pattern of labeling of consecutive sections with NMS was ascertained for comparison. A representative image of a cell incubated with NMS and processed identically to those incubated with immune serum is shown in Figure 6A (arrows in this image point to gold particles). To quantify the proportion of gold labeling that was attributable to the reactivity of the IVI-16/TXNPx antiserum, gold particles in EM sections were enumerated. Figure 6B shows that in sections incubated with the IVI-16/TXNPx antiserum there were approximately33 gold particles/µm^2^ on the parasite, 2 particles/µm^2^ in the PV lumen, 1 particle/µm^2^ in the cytosol and 3.5 particles/µm^2^ in the nucleus. In contrast, there were 1, 0.14, 0.35 and 1.65 gold particles/µm^2^ on the parasite, PV, cytosol and nucleus, respectively, when NMS was applied on these sections. With the exception of the nucleus, the difference between the concentration of gold particles in within cellular compartments of sections incubated with IVI-16/TXNPx antiserum was significantly (p<0.05 t-test) higher than for particles on sections incubated with NMS. Analysis of samples processed after fixation and LR white embedding also showed that the IVI-16/TXNPx antiserum labeled the parasite with a pattern that is best described as cytosolic. In addition, there was evidence of labeling of the PV lumen and vesicles in the cell cytosol outside the PV (Figure 6C) [white arrows point to gold particles and vesicular structures]. The vesicular structures in the cytosol, (some of which are labeled) had a ‘fuzzy’ coat that is reminiscent of coated vesicles. Labeling of such vesicles was observed only with the antiserum to IVI-16/TXNPx. The pattern of labeling of uninfected cells with this antiserum was similar to the pattern of labeling of infected cells with normal mouse serum (NMS).

To address the possibility that the observed labeling in the PV and the infected cell cytosol might be derived from dead parasites, fresh grids containing sections of infected cells were double labeled with the antiserum to the cTXNPx and IVI-16/TXNPx antiserum. A representative image showing the pattern of a double labeled cell is shown in Figure 6D. Reactivity of the cTXNPx was visualized with 5 nm gold particles [thin arrows]. The cTXNPx antiserum resulted in robust labeling of the parasite forming a pattern of labeling that is consistent with a cytoplasmic distribution of the endogenous molecule. The antibody to cTXNPx exhibited minimal reactivity outside the parasite. Labeling with the IVI-16/TXNPx antiserum also resulted in a pattern on the parasite that was consistent with a cytoplasmic distribution pattern. However, it is evident that the IVI-16/TXNPx antiserum labels structures in the PV lumen outside the parasite as well as the host cell cytosol (thick black arrows). Gold particles from such double labeling experiments were enumerated (Figure 6E). There were significantly fewer 5 nm gold particles (cTXNPx) in the PV lumen, the host cell cytosol and nucleus as compared to large gold particles (IVI-16/TXNPx). A surprising observation, however, was that there was less 18 nm gold and 5 nm gold labeling on the parasites in the double labeling experiments than what was observed in the single labeling experiments. This suggested that both antibodies competed for antibody binding sites but only on molecules that were expressed within the parasite. Nonetheless, these results provide additional evidence that the endogenous IVI-16/TXNPx variant is antigenically different from the cytosolic variant previously described. Furthermore, the endogenous IVI-TXNPx molecule appears to traffic out of the parasite into the PV and beyond the PV into the host cell cytosol in coated vesicles. The double labeling experiments argue against the likelihood that labeling in the PV lumen and within the infected cell cytosol was derived from dead parasite material.

**Figure 7:**
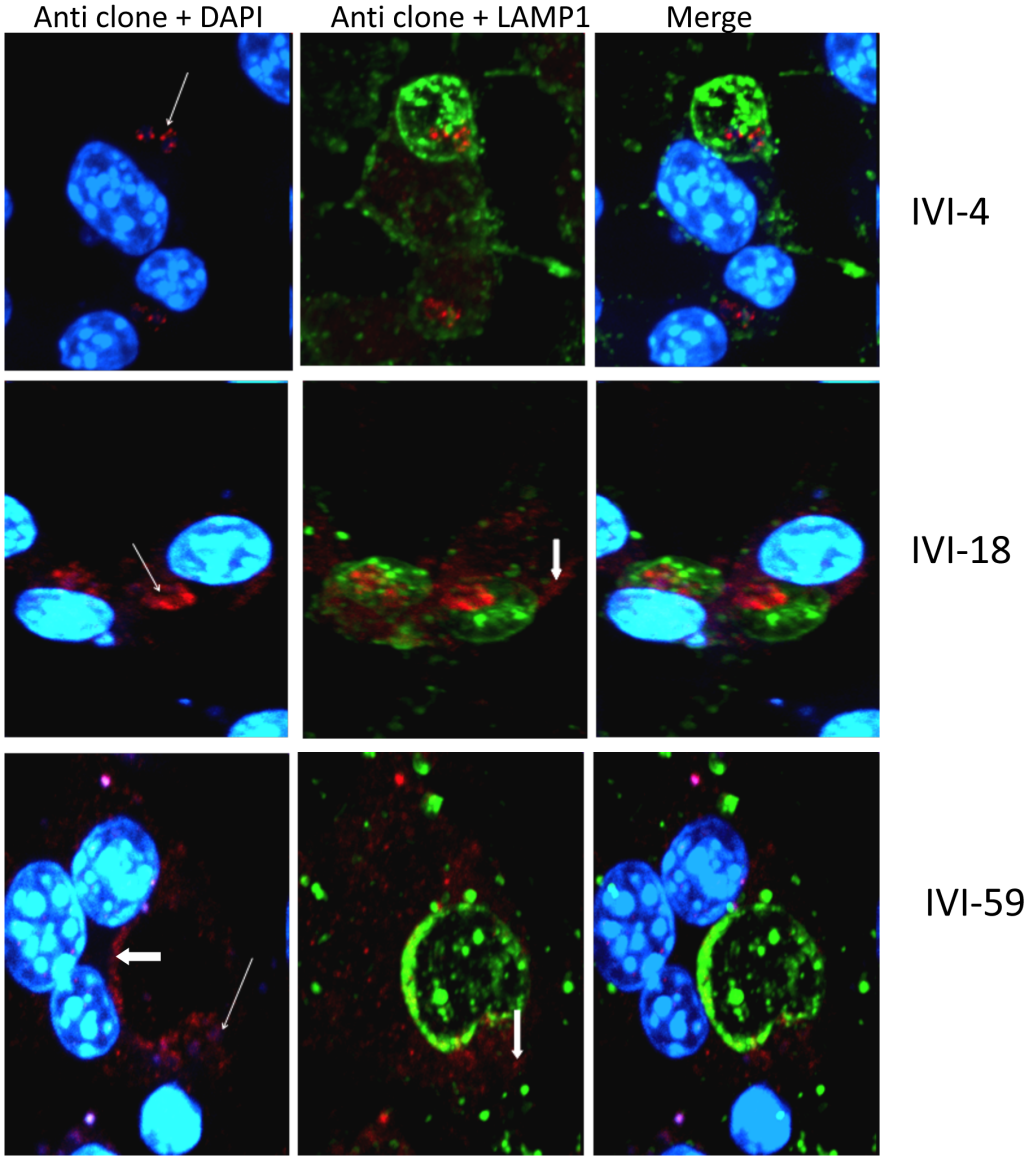
Immunolocalization of the endogenous molecules that are reactive to antisera to IVI-4, IVI-18 and IVI-59. RAW 264.7 macrophages on coverslips were infected for 72 H and then fixed. Immunofluorescence staining was performed with antisera raised to recombinant IVI-4, IVI-18 and IVI-59.

**Figure 8:**
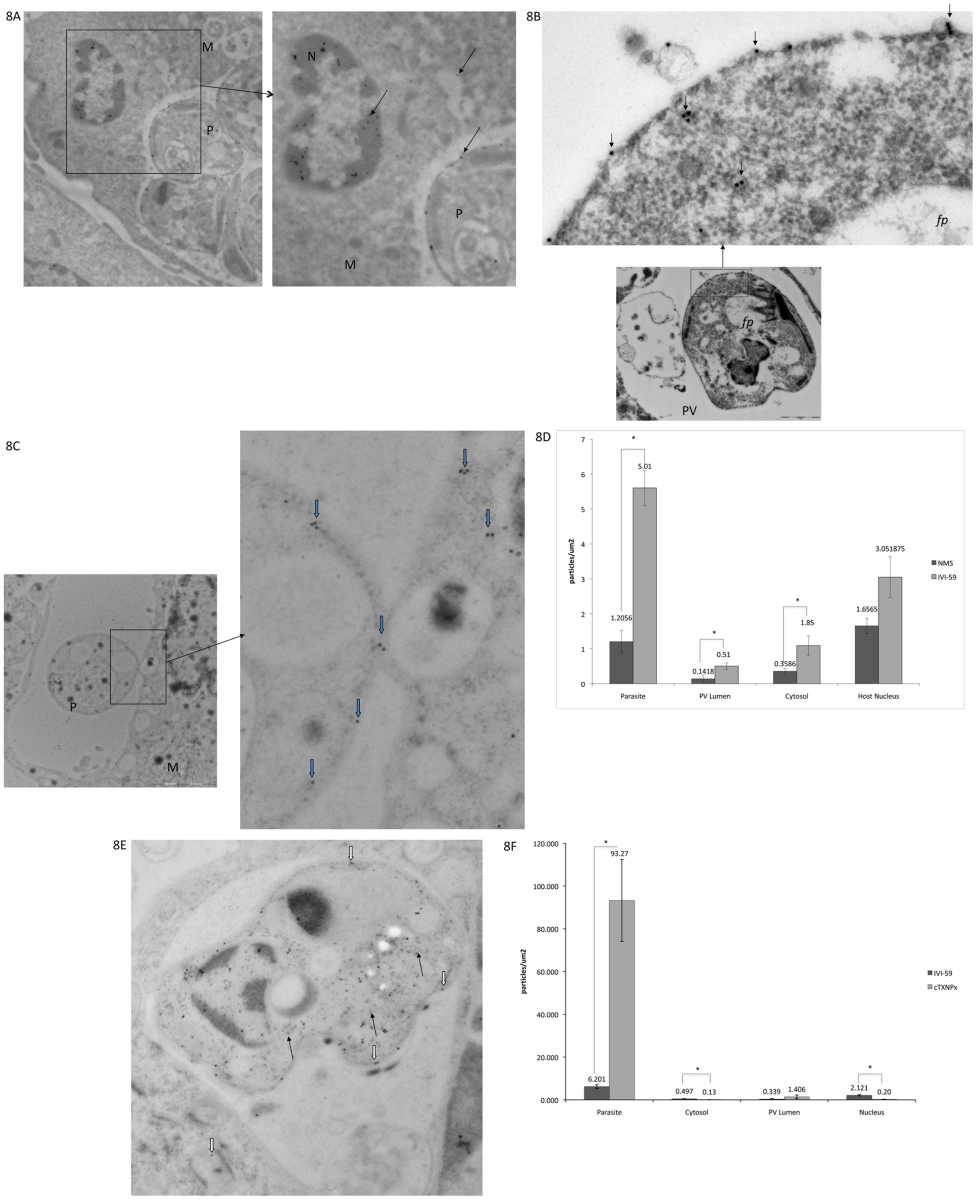
Localization of the endogenous molecule reactive with IVI-59 antiserum by Immuno-EM. A. Infected cells were fixed then embedded in LR white resin. Sections on nickel grids were incubated with the antiserum to IVI-59. This was followed by incubation with an anti-mouse 15 nm-gold conjugated antibody. Labeling of parasites in an infected cell is shown. Highlighted area was blown up to show gold labeling on the parasite surface as well as in the host cell nucleus (thin black arrows point to examples of a few gold particles. The highlighted area was zoomed in to show labeling of the parasite surface and labeling of the host cell nucleus; gold particles were also found in‘empty vesicles’ in the cell cytosol. B. Cryofixed samples embedded in Lowicryl HM20 and sectioned were incubated with antiserum to IVI-59. This was followed by incubation with an anti-mouse 18 nm-gold conjugated antibody. Gold labeling of a parasite in a PV is shown. Arrows point to gold articles in membrane bound structures within the parasite and in vesicular structures on the surface of the parasite in zoomed in images. C. A parasite in a PV is shown; blue arrows point to gold labeling on the parasite surface; labeling at the PV-host cell interface and gold particles in the host cell cytosol. D. Gold particles on sections incubated with either the IVI-59 antiserum or NMS were enumerated and plotted. At least 10 cells were counted per experiment. E. Double labeling experiments; cryofixed samples were incubated with antiserum to IVI-59 (mouse) as well as the antiserum to cTXNPx (rat). The reactivity of cTXNPx was monitored with an anti-rat 5 nm gold conjugated secondary antibody. Thin black arrows point to examples of 5 nm gold; thick black arrows point to examples of 18 nm gold. F. Gold particles in double labeling experiments were enumerated and plotted. Asterix denote significant difference, P<0.5. Legend: M = macrophage; P = Parasite; N = Nucleus.

The distribution of the endogenous molecules that are reactive to antisera to IVI-18 and IVI-59 was also investigated in parallel experiments. Recall that these antisera too were raised to recombinant molecules and that they exhibited no reactivity to macrophage molecules in non-infected cells. Immunofluorescence assays of infected cells with antiserum to IVI-59 showed that this molecule labeled the parasites within PVs (thin white arrow points to representative parasite) (Figure 7). In addition, there was labeling of the PV lumen, PV membrane and structures in the host cell cytosol (bold white arrows). We proceeded to perform immuno-EM analysis to assess further, the reactivity of the IVI-59 antiserum. Sections obtained by LR white embedding of chemically fixed cells showed that the endogenous molecule identified by this antiserum is localized primarily to the parasite surface (Figure 8A [arrows point to examples of gold particles). Unlike the observation made with antiserum to IVI-16/TXNPx that labeled what appeared to be coated vesicles in the host cell cytosol, the IVI-59 antiserum labeled vesicles of various sizes that had no other distinguishing characteristics in the host cell cytosol. We next analyzed sections that were processed after cryofixation. The distribution pattern of the IVI-59 molecule on the parasite was as observed in the chemically fixed and LR White embedded sections. Additional observations were made with the cryofixed samples. One of the more intriguing observations was the presence of gold particles in membrane enclosed compartments within the parasite and also on vesicular structures on the parasite surface (Figure 8B [thin black arrows]). The labeled vesicular structures on the parasite surface appeared to have ‘bubbled’ from the parasite These vesicular structures on the parasite surface are reminiscent of exosomes recently described by Silverman and colleagues [51]. However, these vesicles did not appear to have been released from the flagellar pocket. Another interesting observation with this antiserum was the apparent transfer of the endogenous IVI-59 molecule from parasites in the PV to host cell vesicles (Figure 8C [blue arrows point to gold particles on the parasite surface, at the parasite-macrophage interface and in vesicular structures within the host cell]). The recipient vesicle in the host cytosol had similar characteristics to other vesicles of various sizes with no other outstanding characteristics that were labeled with gold particles in the host cell cytosol. Finally, immuno-labeling of these sections also exhibited reactivity of the host cell nucleus. Gold particles on the parasite, the PV lumen, the host cell cytosol and the host cell nucleus were enumerated and compared to the distribution of gold particles on sections that were incubated with NMS. Figure 8D shows that there was significantly more gold particles in all the compartments analyzed when the sections were incubated with the antiserum to IVI-59. Double labeling experiments with the IVI-59 antiserum and the antiserum to cTXNPx were also performed; a representative image of a double labeled cell is shown in Figure 8E. The cTXNPx exhibits robust labeling of the parasite (black arrows). In contrast the IVI-59 antiserum primarily labels the parasite surface (bold white arrows). The concentration of gold particles on these sections was determined and plotted (figure 8F). There was considerable labeling of the parasite by the antiserum to cTXNPx but minimal reactivity elsewhere. Interestingly there was no apparent interference between the cTXNPx labeling and the labeling with the IVI-59 antiserum as was noted with the IVI-16/TXNPx antiserum. Taken together, analysis of the distribution of the endogenous molecule (homolog of LinJ31_V3.1500) that is reactive with IVI-59 antiserum showed that this molecule is expressed primarily on the parasite surface. However, it is released into infected cells and traffics outside of the PV to the host cell nucleus. Here too, the double labeling experiments suggested that the extra-parasite labeling of the IVI-59 antiserum is unlikely to be the result of trafficking of molecules from dead parasites.

**Figure 9:**
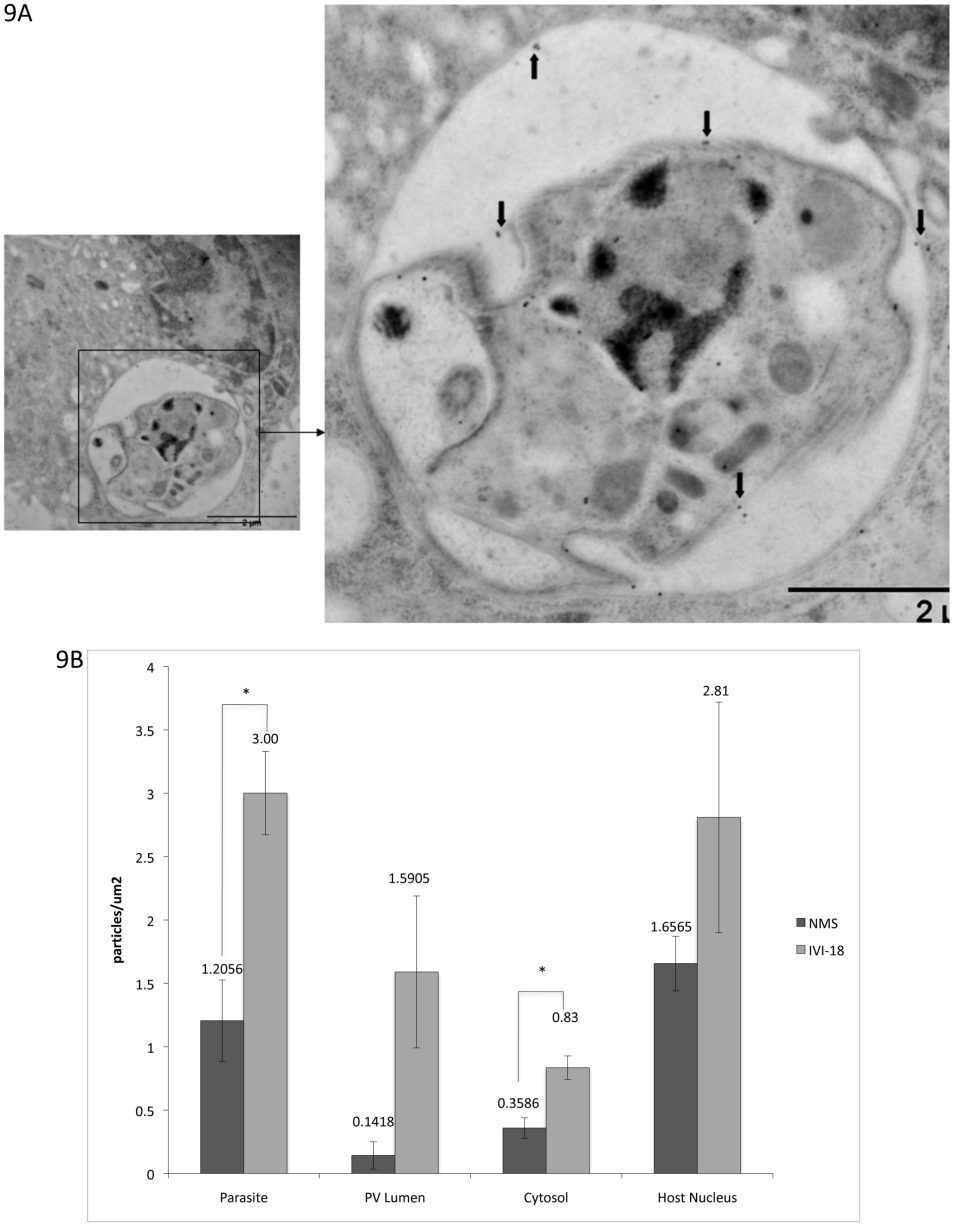
Immunolocalization of the endogenous molecule that is reactive to IVI-18 antiserum. A. Cryofixed samples embedded in Lowicryl HM20 and sectioned were incubated with antiserum to IVI-18. This was followed by incubation with an anti-mouse 18 nm-gold conjugated antibody. Gold labeling of a parasite in a PV is shown. Arrows point to gold particles on the parasite surface. B. Gold particles on sections incubated with either the IVI-18 antiserum or NMS were enumerated and plotted. At least 10 cells were counted per experiment. The difference between counts from the specific antiserum and NMS were assessed in a t-test. Asterix denote significant difference, P<0.5.

The distribution of the IVI-18 molecules was also analyzed in parallel experiments. Figure 7 shows a representative image of an infected cell captured after immunofluorescence staining with antiserum to IVI-18. There is specific staining of parasites within infected cells. We proceeded to analyze samples processed for immuno-EM analyses. There was no difference of interest between samples processed by the chemical fixation and LR white embedding protocol and the cryofixed samples. A representative image from cryofixed samples shows the distribution pattern of the endogenous molecule recognized by the IVI-18 antiserum (Figure 9A [black arrows point out representative gold particles]). This molecule appears to be expressed primarily on the parasite surface. Gold particles on the parasite, PV lumen, host cell cytosol and host cell nucleus were enumerated and plotted. When sections were incubated with the IVI-18 antiserum, the number of gold particles on parasites was significantly higher than that obtained with sections incubated with NMS (Figure 9B). There were also more gold particles in the other compartments analyzed as compared to incubation with NMS. It is noteworthy that the overall concentration of gold particles in sections incubated with the IVI-18 antiserum was less than what was obtained with the IVI-59 antiserum, for example. Taken together, these observations too show that the homolog of LmjF30.2390 that is recognized by the antiserum to IVI-18 is primarily localized to the parasite surface but that it also gains access to the host cell cytosol and nucleus.

The reactivity of the antiserum raised to the IVI-4 was unique as compared to the other CMAT molecules. Although there was strong reactivity to parasites within PVs there was minimal reactivity of this antiserum elsewhere in the infected cell (Figure 7). This suggested that the homolog of LmjF25.0450 identified by IVI-4 is neither released into the PV nor secreted into the host cell cytosol. Representative confocal images of the reactivity of antisera to IVI-62, IVI-63 and IVI- 64 are included in the supplemental figures (Figure S3). Both IVI-63 and IVI- 64 appear to be localized to the surface of parasites within PVs. These observations confirm previous reports that had shown that the AMA molecule (homolog of IVI-63) and some amastin variants (IVI-64) are expressed on the parasite's surface [49]. Table 2 shows a summary of the distribution of the endogenous molecules identified in this study.

**Table 2:** Cellular location of CMAT molecules.*

Molecule (clone)	PV	Host cell cytosol	Host cell nucleus
IVI-4	−/+ (punctuate labeling of parasite)	-	-
IVI-16/aTXNPx	+	++ (fuzzy vesicle)	+/−
IVI-18	+	+ (‘empty vesicles’)	+
IVI-59	+ (strong parasite surface labeling)	+ (‘empty vesicles’)	++ (localization to peripheral heterochromatin)
IVI-62 (cysteine proteinase)	+	++	+/−
IVI-64 (amastin-like surface protein)	+ (parasite surface labeling	+	++ (localization to heterochromatin, not in nuclear periphery)

*Data was obtained from analyses of IFA's and immuno-EM.

### Nuclear localization of exported IVI molecules

**Figure 10:**
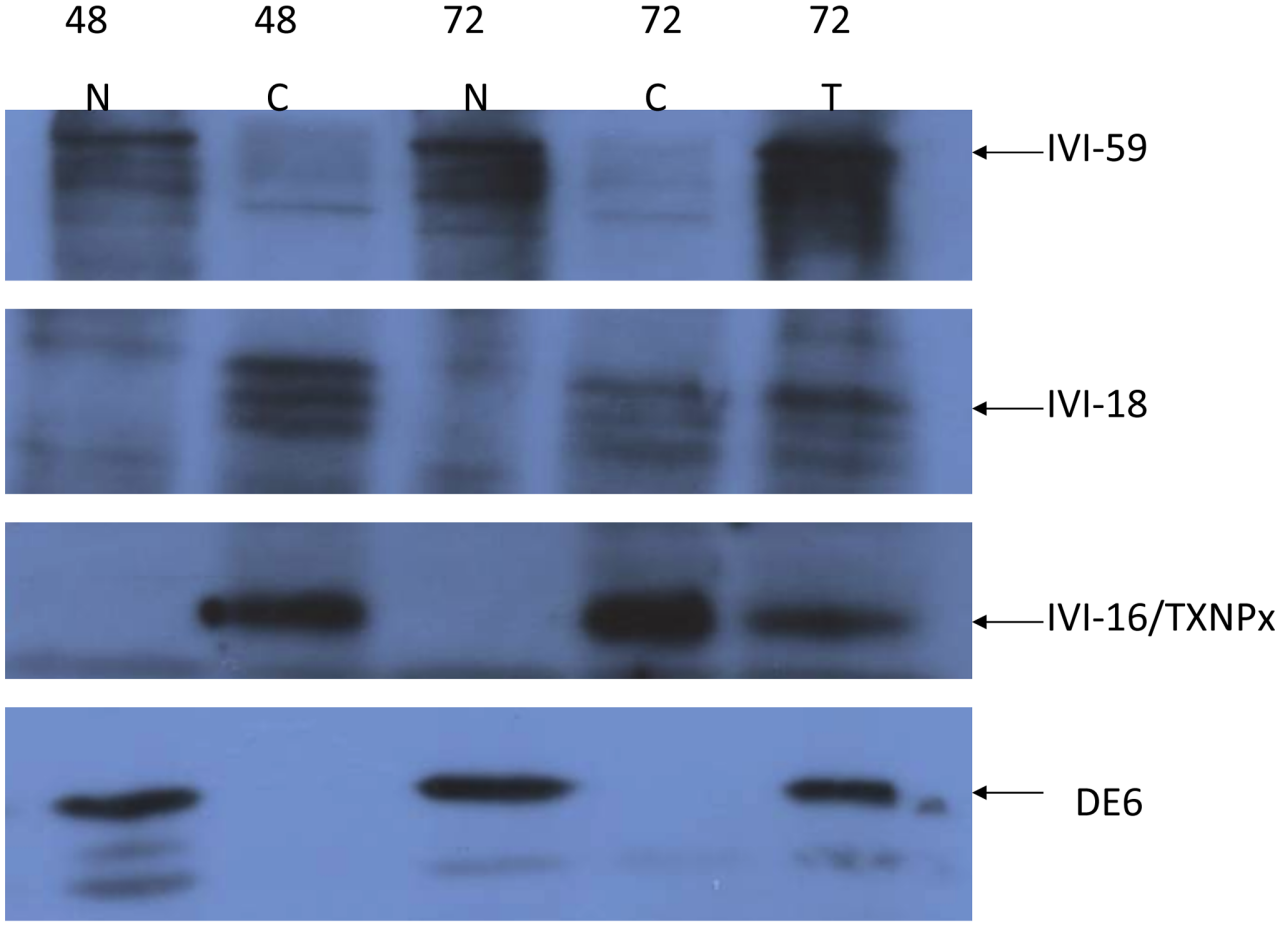
Localization of IVI molecules in nuclear and cytosolic fractions of infected cells. Macrophages infected for 48 or 72 H were recovered cytosolic and nuclear fractions were prepared. Equal protein aliquots of the total lysate (T) and each fraction was run on 12% PAGE and blotted. Identical blots were probed with antisera to IVI-16/TXNPx, IVI-18 or IVI-59. The blots were stripped and probed with DE6, which reacts to a nucleolar protein. The region of each gel with the expected bands was cropped and combined to make the composite figure. This experiment was run twice.

The localization studies of the endogenous IVI molecules described above suggested that some of those parasite molecules might traffic to the host cell nucleus. To determine whether endogenous IVI molecules are localized in the nucleus of infected cells, a subcellular fractionation protocol to isolate the nucleus of infected cells was implemented. The resulting nuclear and cytosolic fractions were analyzed by Western blot analysis for their reactivity with antisera to IVI-16/TXNPx, IVI-18 and IVI-59. After 48 and 72 H infections, the IVI-16/TXNPx antiserum is exclusively reactive with the cytosolic fraction (Figure 10). Similarly, the antiserum to IVI-18 is mostly reactive with the cytosolic fraction with occasional faint reactivity with the nuclear fraction. In contrast, the IVI-59 antiserum is reactive with both the nuclear and cytosolic fractions. Its reactivity with the nuclear fraction is with a molecule that is at the appropriate molecular weight. The quality of the subcellular fractionation was monitored with the reactivity of DE6, an antibody that is specific for a 47/51 kDa nucleolar protein [52]. Taken together, these observations complement the results obtained by counting gold particles on EM sections that had found that the labeling of the nucleus with the IVI-59 antiserum was significantly more than that obtained with NMS. In contrast although there was more gold labeling of the host nucleus with antisera to IVI-16/TXNPx and IVI-18, the difference was not significantly different from the labeling with NMS. An important caveat in the interpretation of these results though is that the subcellular fractionation protocol as implemented was stringent and could therefore exclude some molecules that traffic to the nucleus under physiological conditions.

Prior to the observation reported here of *Leishmania* parasite molecules trafficking in the infected cell, elongation factor 1α (EF-1α) from *Leishmania* had been shown to localize to the host cell cytosol where it targets SHP-1 [53], [54]. EF-1α was identified within macrophages after 16 hours of infection by promastigotes, however the mechanism by which EF-1α accesses the host cell cytoplasm has not been described. Several lines of evidence have also suggested that cysteine proteinases too, must gain access to the infected cell cytosol where they target components of the NFκB signaling complex [55]. This led Mottram and colleagues to propose that cysteine proteinases are most likely transported out of the PV in vesicles which rupture in the cytosol [56]. In this study, IVI-62 (a cysteine proteinase homolog) was identified by the CMAT screen and found to gain access to the cell cytosol. In addition to IVI-62, several lines of evidence were obtained that showed that other *Leishmania* parasite molecules are released beyond the PV as well.

It is noteworthy that the presence or absence of a signal sequence was not predictive of the trafficking scheme of parasite molecules in the infected cell. This observation is in agreement with a recent study in which parasites molecules that are secreted into culture medium (secretome) were analyzed [57]; in that study, it was concluded that most of the molecules identified did not appear to be secreted through the classical secretion pathway. The authors discussed that *Leishmania* might possess several secretion pathways in addition to secretion through the flagellar pocket. In this study, the vesicular structures that were reactive with the IVI-59 antiserum might be a part of one of those secretion pathways. Further studies employing reagents to the parasite molecules identified in this study should provide greater insight into protein secretion by *Leishmania* parasites within infected cells.

The protein products of the CMAT genes analyzed thus far were detected in infected cells after many hours (days) of infection, however, their transcripts were detected in parasites that were cultured axenically (not shown). Studies on LIT1, the ZIP family iron transporter had also found that although the transcripts of LIT1 are found in promastigotes and axenic amastigotes, the protein product is detected only in parasites within PVs after several days of infection [12]. Together, these observations suggest that of the estimated 8300 *Leishmania* proteins there is a subset of genes for which the protein products are synthesized only in the intracellular environment. This subset might also include parasite molecules that are preferentially expressed at discrete times in the infected host. It is not known how many molecules would fall into this subset. Such molecules are of great interest because they might mediate the pathogenic mechanisms of the parasite and represent biomarkers of disease progression in the infected host.

CMAT, like IVIAT, has limitations that should be acknowledged. It is an immunoscreen so only molecules that are immunogenic will be identified; this implies that molecules that undergo lipid or carbohydrate modifications in response to the environmental changes in the PV may not be detected by these methods. In addition, genetic control of immune reactivity can limit the diversity of epitopes that are generated in a given host. The later limitation can be minimized by using outbred animals (hamsters and rabbits) for the generation of antisera; alternatively, pooled human convalescence sera can be obtained. The expression library constructed in pET expression vectors might not be fully representative as it relies on the availability of Sau3A sites which might not be in the proximity of some genes. Lastly, as implemented here, the approach is dependent on significant difference in gene expression between axenically cultured parasites and parasites that grow within cells.

There might be some benefit to using established parasite lines or parasite lines that have undergone multiple passages in axenic culture as was done in this CMAT approach. The rationale that is often stated for using parasites soon after they are recovered from the *in vivo* environment is that they might still be expressing *in vivo* induced pathogenic molecules. How soon the expression of such molecules is suppressed is however not known. If parasites that were recently established in culture were used in CMAT, the continued presence of *in vivo* expressed molecules might result in the removal of antibodies that are reactive to them during the adsorption protocol. Since it is not known which molecules have primary functions in the intracellular environment, it would be impossible to determine what has been lost.

## Supporting Information

Figure S1 The phylogenetic relationship between the TXNPxs. The phylogenetic relationship between the TXNPxs was inferred using the Neighbor-Joining method. The percentage of replicate trees in which the associated taxa clustered together in the bootstrap test (1000 replicates) are shown next to the branches. The tree is drawn to scale, with branch lengths in the same units as those of the evolutionary distances used to infer the phylogenetic tree. The evolutionary distances were computed using the Poisson correction method and are in the units of the number of amino acid substitutions per site. All positions containing gaps and missing data were eliminated from the dataset (complete deletion option). There was a total of 175 positions in the final dataset. Phylogenetic analyses were conducted in MEGA4.
(0.50 MB TIF)

Figure S2 Sequence comparison of the CMAT clone 64 sequence with amastin-like sequences in the GeneDB genome database and phylogenetic analysis. The sequence in CMAT clone 64 was aligned with genes in the GeneDB genome containing amastin-like sequences. B) The phylogenetic relationship was inferred using the Neighbor-Joining method [58]. The percentage of replicate trees in which the associated taxa clustered together in the bootstrap test (1000 replicates) are shown next to the branches. The tree is drawn to scale, with branch lengths in the same units as those of the evolutionary distances used to infer the phylogenetic tree. The evolutionary distances were computed using the Poisson correction method and are in the units of the number of amino acid substitutions per site. All positions containing gaps and missing data were eliminated from the dataset (complete deletion option). There was a total of 101 positions in the final dataset. Phylogenetic analyses were conducted in MEGA4 [59].
(1.74 MB TIF)

Figure S3 Immunolocalization of native molecules identified by CMAT in infected cells. RAW 264.7 macrophages on coverslips were infected for 72 hours and then fixed. Immunofluorescence staining was performed with antisera raised to recombinant IVI molecules shown. LAMP1(green label) reactivity identifies lysosomes and delineates the PV membrane. Specific reactivity by each antiserum is in red. The thin arrow points to a representative parasite in a PV. The thick arrow points to an example reactivity outside PVs. These images were captured with a Zeiss confocal microscope integrated into a spinning disc technology system from PerkinElmer; Z stack series were combined with the extended focus feature in the velocity software. Immunofluorescence assays are representative of more than 3 experiments with these antisera.
(2.38 MB TIF)
