# Supplementary figures and images for: Identification of Leishmania Proteins Preferentially Released in Infected Cells Using Change Mediated Antigen Technology (CMAT)
Source: PLoS Negl Trop Dis. 2010 Oct 5;4(10):e842. doi: 10.1371/journal.pntd.0000842 (PMC2950143; doi:10.1371/journal.pntd.0000842)

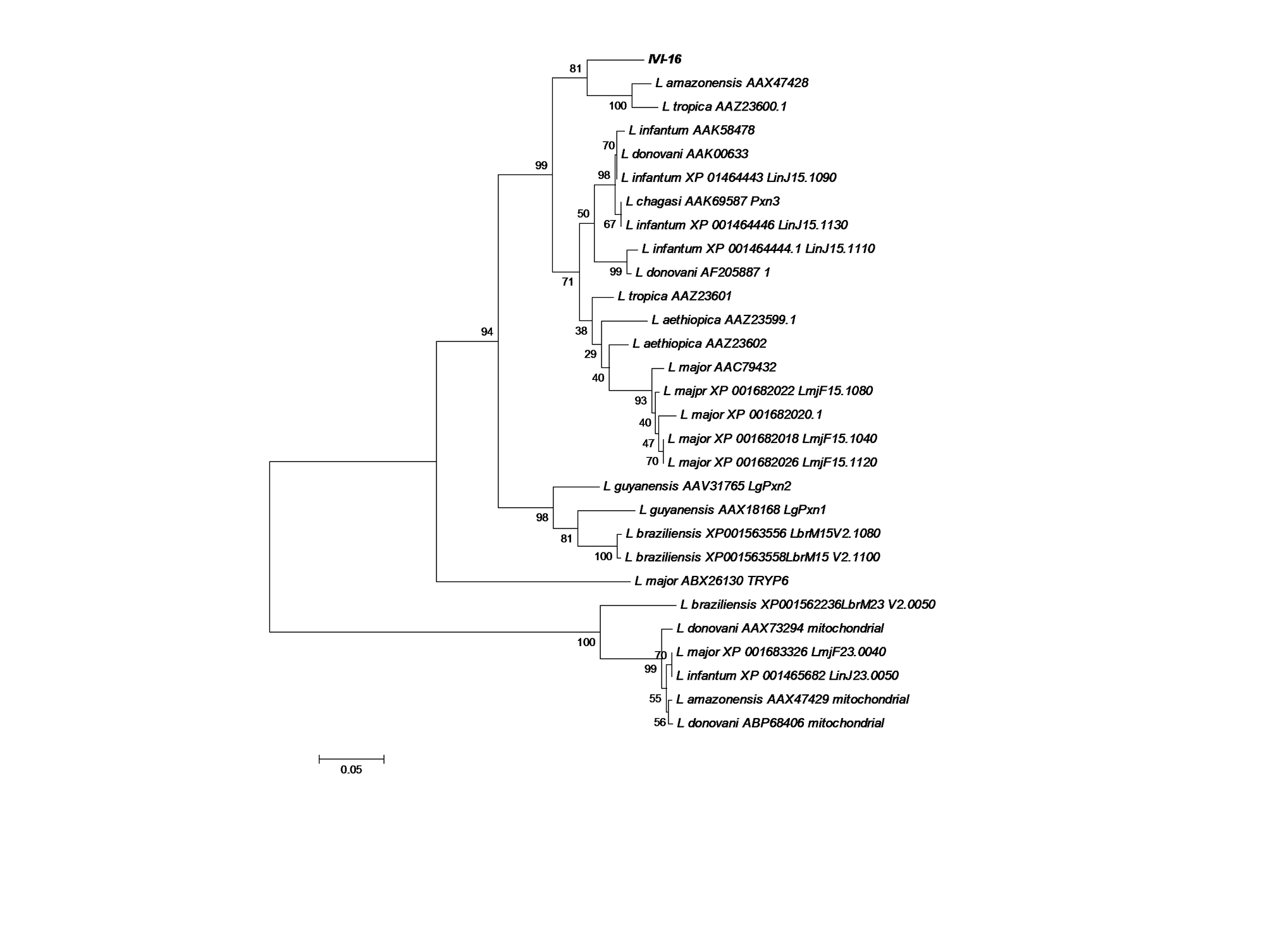

Supplement: Figure S1 — The phylogenetic relationship between the TXNPxs. The phylogenetic relationship between the TXNPxs was inferred using the Neighbor-Joining method. The percentage of replicate trees in which the associated taxa clustered together in the bootstrap test (1000 replicates) are shown next to the branches. The tree is drawn to scale, with branch lengths in the same units as those of the evolutionary distances used to infer the phylogenetic tree. The evolutionary distances were computed using the Poisson correction method and are in the units of the number of amino acid substitutions per site. All positions containing gaps and missing data were eliminated from the dataset (complete deletion option). There was a total of 175 positions in the final dataset. Phylogenetic analyses were conducted in MEGA4. (0.50 MB TIF) [file pntd.0000842.s001.tif]

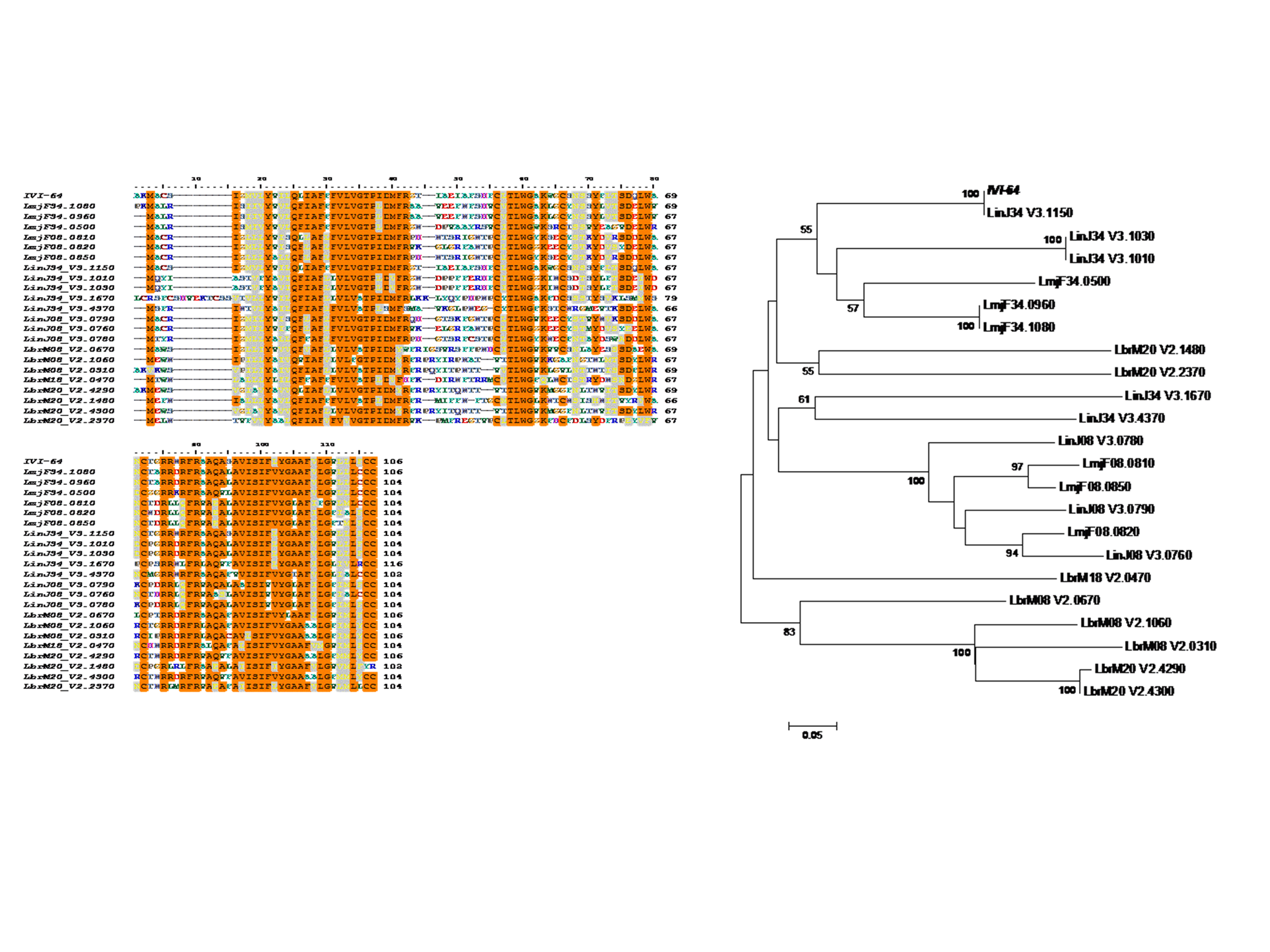

Supplement: Figure S2 — Sequence comparison of the CMAT clone 64 sequence with amastin-like sequences in the GeneDB genome database and phylogenetic analysis. The sequence in CMAT clone 64 was aligned with genes in the GeneDB genome containing amastin-like sequences. B) The phylogenetic relationship was inferred using the Neighbor-Joining method [58]. The percentage of replicate trees in which the associated taxa clustered together in the bootstrap test (1000 replicates) are shown next to the branches. The tree is drawn to scale, with branch lengths in the same units as those of the evolutionary distances used to infer the phylogenetic tree. The evolutionary distances were computed using the Poisson correction method and are in the units of the number of amino acid substitutions per site. All positions containing gaps and missing data were eliminated from the dataset (complete deletion option). There was a total of 101 positions in the final dataset. Phylogenetic analyses were conducted in MEGA4 [59]. (1.74 MB TIF) [file pntd.0000842.s002.tif]

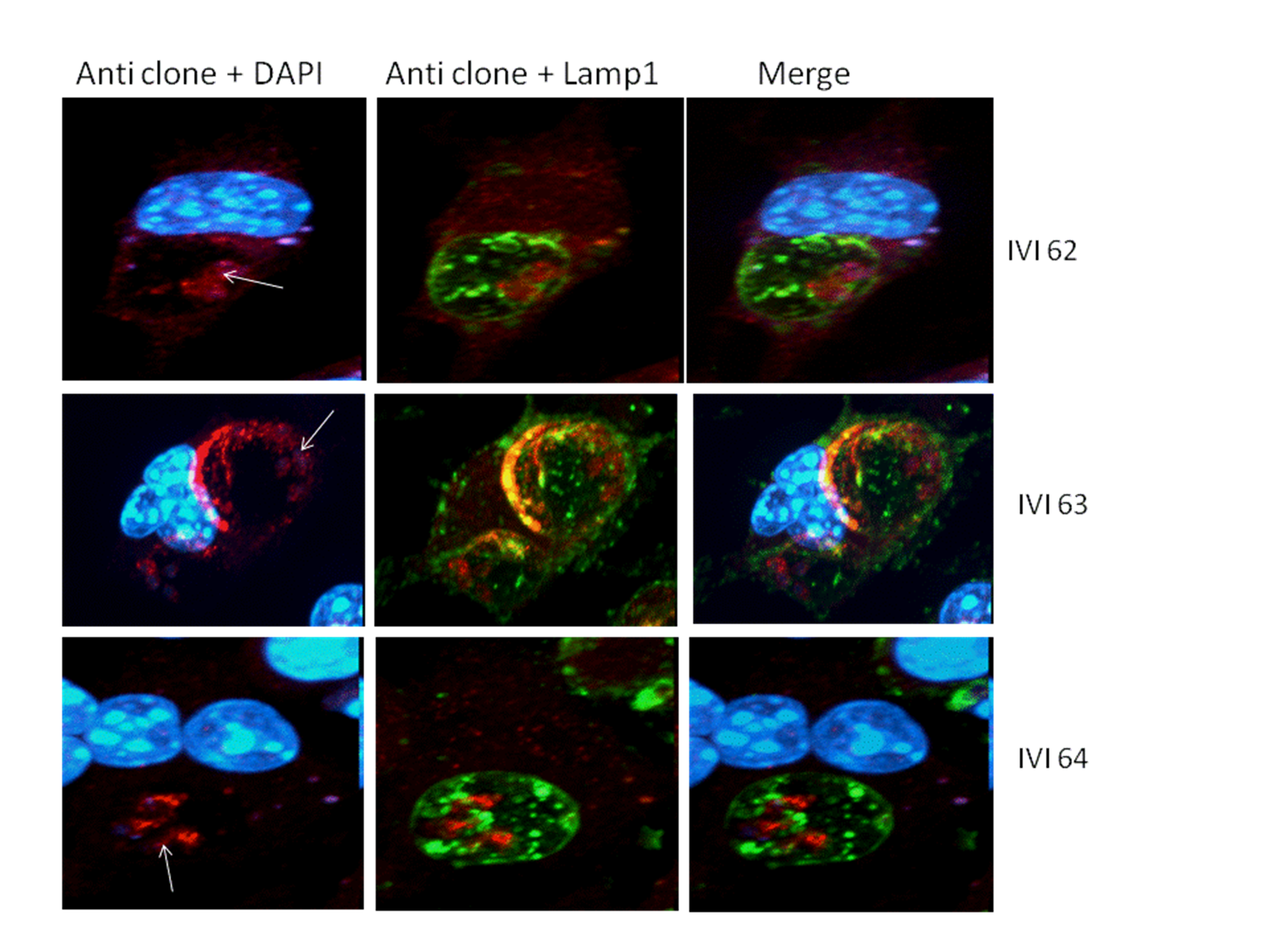

Supplement: Figure S3 — Immunolocalization of native molecules identified by CMAT in infected cells. RAW 264.7 macrophages on coverslips were infected for 72 hours and then fixed. Immunofluorescence staining was performed with antisera raised to recombinant IVI molecules shown. LAMP1(green label) reactivity identifies lysosomes and delineates the PV membrane. Specific reactivity by each antiserum is in red. The thin arrow points to a representative parasite in a PV. The thick arrow points to an example reactivity outside PVs. These images were captured with a Zeiss confocal microscope integrated into a spinning disc technology system from PerkinElmer; Z stack series were combined with the extended focus feature in the velocity software. Immunofluorescence assays are representative of more than 3 experiments with these antisera. (2.38 MB TIF) [file pntd.0000842.s003.tif]
